# Time-course RNA-seq analysis of upland cotton (*Gossypium hirsutum* L.) responses to Southern root-knot nematode (*Meloidogyne incognita*) during compatible and incompatible interactions

**DOI:** 10.1186/s12864-025-11339-w

**Published:** 2025-02-24

**Authors:** Sameer Khanal, Pawan Kumar, Mychele B. da Silva, Rippy Singh, Nelson Suassuna, Don C. Jones, Richard F. Davis, Peng W. Chee

**Affiliations:** 1 Department of Crop and Soil Sciences and Institute of Plant Breeding, Genetics, and Genomics, University of Georgia, 2356 Rainwater Road, Tifton, GA 31793 USA; 2 Bayer Crop Science Division, 700 W. Chesterfield Pkwy W, Chesterfield, MO 63017 USA; 3 Tidewater AREC, Virginia Tech, 6321 Holland Road, Suffolk, VA 23437 USA; 4 Empresa Brasileira de Pesquisa Agropecuária (Embrapa), Santo Antônio de Goiás, GO Brazil; 5 Cotton Incorporated, 6399 Weston Parkway, Cary, NC 27513 USA; 6 U.S. Department of Agriculture – Agricultural Research Service, 115 Coastal Way, Tifton, GA 31793 USA

**Keywords:** Comparative transcriptomics, Host-parasite interaction, Host plant resistance, Differentially expressed genes and pathways, Cotton molecular breeding

## Abstract

**Background:**

The Southern root-knot nematode (*Meloidogyne incognita*) poses a substantial threat to cotton (*Gossypium hirsutum* L.) by causing significant agricultural losses. Host plant resistance is the most plausible approach for minimizing these losses. QTL mapping and early transcriptomic studies have identified candidate genes within the QTL regions on chromosome 11 (*qMi-C11*) and chromosome 14 (*qMi-C14*). Although these QTL regions have been fine-mapped and candidate genes identified, expression profiling of *Meloidogyne-Gossypium* interactions across different stages of infection could further refine the list of candidate genes. This study advances our understanding of the molecular mechanisms underlying the resistance conferred by *qMi-C11* and *qMi-C14* against Southern root-knot nematode.

**Results:**

Using time-course RNA-seq analyses across nematode developmental phases, we uncovered transcriptomic events—both genome-wide and within QTL intervals—underlying defense responses during compatible interactions (with Cocker 201, a susceptible line) and incompatible interactions (with M-120 RNR, a resistant line). Basal defense responses were observed in both compatible and incompatible interactions, with stronger expression in the incompatible interaction. Nematode-responsive genes associated with defense pathways showed distinct dynamics, characterized by repression during compatible interactions and early induction, greater diversity, and heightened upregulation during incompatible interactions. This study uncovers a broad repertoire of disease resistance and putative resistance genes, as well as pathogenesis-related genes, ligands, and receptors, that are differentially expressed in response to nematode parasitism. Mapping of these genes across the cotton genome identified promising candidates, including Gh_A11G3090 (*PUB21*) and Gh_A11G2836 (*RPPL1*) within the chromosome 11 QTL region, andGh_D02G0257 (*RLP12*) and Gh_D02G0259 (*RLP12*) within the chromosome 14 QTL region.

**Conclusions:**

The findings of this study deepen our understanding of host-nematode interactions, identify candidate genes for downstream applications, and contribute to advancements in resistance breeding and sustainable nematode management strategies.

**Supplementary Information:**

The online version contains supplementary material available at 10.1186/s12864-025-11339-w.

## Background

The Southern root-knot nematode (RKN), *Meloidogyne incognita*, is one of the most important agricultural pests of upland cotton [*Gossypium hirsutum* L.; 2n = 4x = 52 (subgenomes ‘A’ and ‘D’ with 26 chromosomes each) due to its widespread distribution, genetic diversity, broad host range, and polyphagous feeding habits [1, 2]. In the United States alone, *M. incognita* has caused losses exceeding 65 million kilograms of cotton, valued at over 123 million dollars [3, 4]. Recommended management practices include cultural controls, such as crop rotation and cover crops, biological control with nematopathogenic bioagents, and chemical control through nematicide applications [5]. However, the broad host range of *M. incognita* complicates cultural and biological management methods, leaving growers with limited nonhost crop options for rotation. Nematicides, while effective for early-season nematode suppression, significantly increase production costs.

Host plant resistance is the most economical, practical, and environmentally sound method for managing this subterranean pest. Currently, the resistant line ‘Auburn 623 RNR’ and the elite breeding ‘M’ lines derived from it remain the primary source of RKN resistance. Histopathological comparisons of compatible and incompatible interactions—resulting in successful infection or defensive response respectively—using M-lines or their derivatives have shown that resistance operates through a two-stage post-penetration interference. The first, or ‘early’ mechanism occurs soon after infection (8–12 days after inoculation) to prevent juvenile nematodes from developing functional feeding sites. The second, or ‘late’ mechanism, occurs later in the infection process (25–30 days after inoculation) to impede the development of nematodes into egg-laying adult females [6–10]. These findings were supported by molecular genetic studies, which identified two QTLs, *qMi-C11* and *qMi-C14*, that confer RKN resistance. The *qMi-C11* region suppresses root gall formation, while *qMi-C14* reduces egg production with minimal impact on galling [11–15]. Resistance conferred by *qMi-C14* has been attributed to the gene Gh_D02G0276, which encodes for hAT-like transposases with novel N- and C-terminal domains that resemble targets of known RKN effector molecules [16]. However, the causal gene underlying *qMi-C11* remains unidentified.

Comparative transcriptomic analysis using time-course RNA sequencing (RNA-seq) data enables researchers to pinpoint candidate genes within QTL regions and uncover genome-wide, spatiotemporal expression dynamics of genes involved in complex regulatory and biochemical pathways. This approach has been successfully applied to identify nematode-responsive genes and pathways involved in host-nematode interactions. Time-course RNA-seq has been used to elucidate RKN-host interactions in plant families such as Fabaceae [17], Rosaceae [18], Cucurbitaceae [19, 20], and Solanaceae [21, 22]. Comparative genomics has also been used as a predictive tool to identify conserved plant genes responsive to RKN infections [23]. The resistance mechanisms observed in the RKN-resistant lines derived from Auburn 623 RNR are similar to those found in *Cucumis metuliferus* [20], *Vigna unguiculata* [24], and *Medicago truncatula* [25], where resistance does not prevent nematode penetration and does not involve a hypersensitive response (HR). Comparative host-RKN transcriptomics, using data from diverse plant species exposed to RKN, offers great potential for identifying candidate genes involved in host-RKN interactions. This approach is facilitated by advancements in whole genome sequencing, which have improved our understanding of synteny and collinearity among plant genomes.

In an exploratory RNA-seq study, numerous differentially expressed genes were identified in a susceptible cotton line (compatible interactions) and its near-isogenic line with nematode resistance (incompatible interactions) 12 and 30 days after inoculation [26]. Two key differences in gene regulation were observed between the early and late stages of infection. First, the resistant genotype induced the expression of genes to resist invasion and restrict the movement of RKN within the roots, whereas the susceptible genotype expressed genes that facilitated the establishment of feeding sites and the development of female RKN. Second, while the QTL loci may contribute to RKN resistance, the major contribution likely stems from the elevated expression of defense response genes common to both genotypes. An RNA-seq study conducted 10 days-after-inoculation supports this observation, revealing that defense response genes regulated by hormones such as jasmonic acid (JA) and salicylic acid (SA) are constitutively expressed but accumulate at higher levels in the roots of the resistant genotype ‘Acala NemX’ [27]. Thus, key genetic switches controlling resistance need to be investigated by examining gene expression across various stages of infection, from the establishment of feeding sites by the *M. incognita* second-stage juveniles (J2s) to their postinfection development into adult females.

We conducted a comprehensive time-course RNA-seq analysis using the resistant and susceptible genotypes and experimental conditions reported by Kumar et al. [26], tracking the entire life cycle of *M. incognita* development in cotton roots [26]. Comparative transcriptomic analysis was performed across five developmental phases (4, 8, 12, 16 and 20 days after inoculation, DAI), providing a clearer understanding of the transcriptomic changes and molecular mechanisms underlying the two modes of resistance conferred by *qMi-C11* and *qMi-C14* QTLs. This study lays the groundwork for narrowing down the list of candidate genes previously identified for these QTLs and deepens our understanding of the molecular mechanisms involved in host-plant interaction.

## Results

### Transcriptome sequencing and mapping

A total of 44 RNA-seq libraries were sequenced. After trimming adapters and removing low-quality reads, the number of reads per library ranged from 8.75 to 20.30 million, with an average of 12.98 million. Of the 574.2 million total reads, 483.99 million (84.28%) were mapped to the reference 'TM-1' genome [28] (Supplemental Figure S1), obtained from CottonGen database (www.cottongen.org) [29].

### Differential expression of genes and enriched categories

**Fig. 1:**
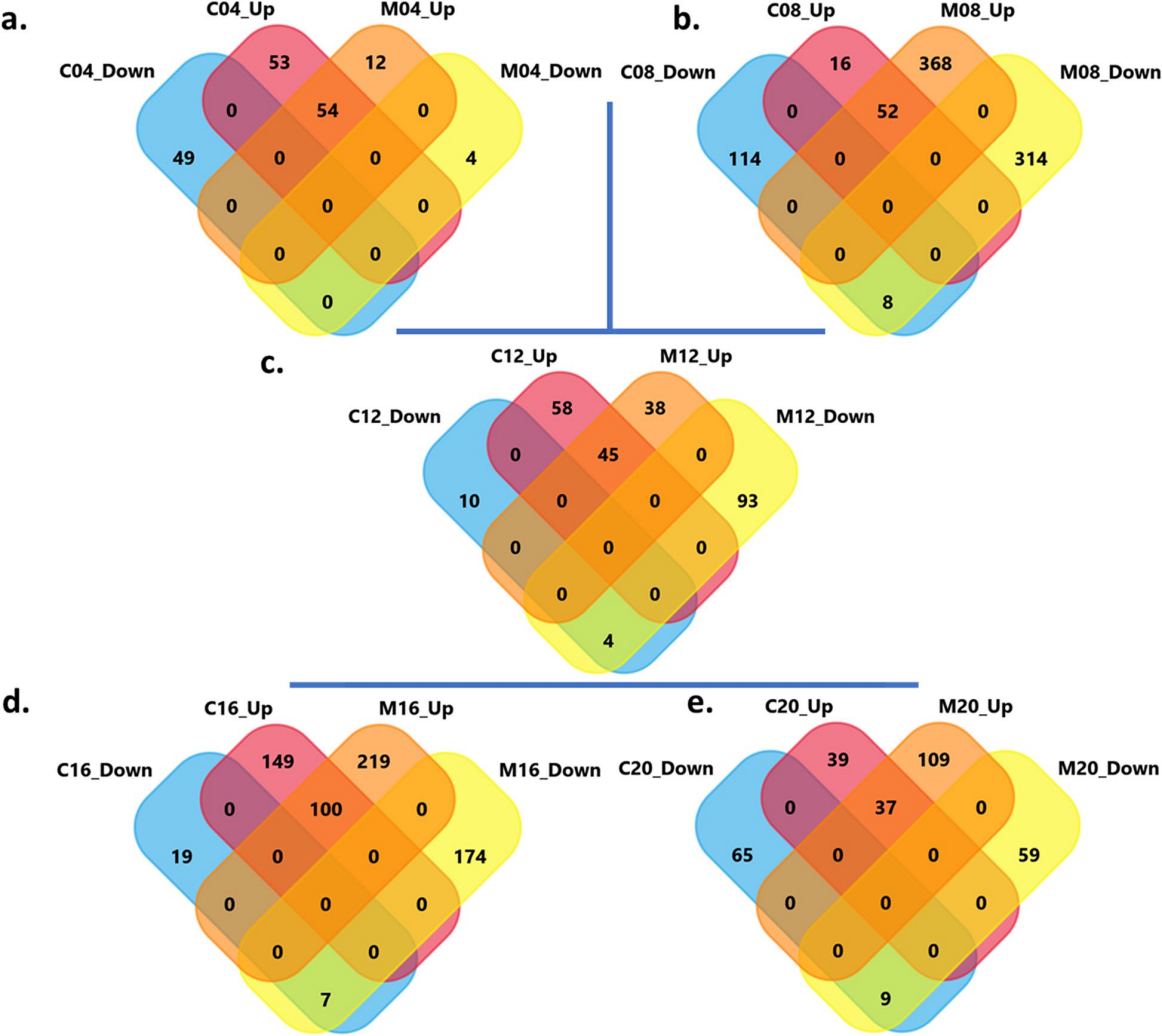
Venn diagrams of the number of DEGs during compatible and incompatible interactions. Diagrams show compatible (susceptible, C201) and incompatible (resistant, M120) interactions between cotton and southern root-knot nematode at a. 4 days after infection (DAI), b. 8 DAI, c. 12 DAI, d. 16 DAI and e. 20 DAI. ‘C’ and ‘M’ correspond to C201 and M120, respectively. Numbers following ‘C’ and ‘M’ correspond to different DAI. ‘Up’ and ‘Down’ correspond to upregulated and downregulated

A total of 2,471 differentially expressed genes (DEGs), corresponding to 1,674 unique genes, were found to be significant (FDR < 0.05; log_2_ FC ≤ −2 or ≥ + 2) in RKN-treated plants compared to the corresponding untreated controls at five different time points (Table 1, Supplemental Table S1). In response to nematode parasitism, incompatible interactions exhibited twice as many DEGs (1,662) as compatible interactions (809), with upregulated genes comprising 60-70% of both interaction types. The highest number of DEGs during incompatible interactions occurred at 8 DAI (724), followed by 16 DAI (493). Venn diagrams illustrate the number of DEGs across the five time points (Fig. 1).

**Table 1:** Number of DEGs during compatible and incompatible interactions between cotton and southern root-knot nematode

Genotype	Contrasts^a^	Downregulated	Upregulated	Total
C201	4 DAI RKN vs. 4 DAI Control	43	105	148
	8 DAI RKN vs. 8 DAI Control	117	63	180
	12 DAI RKN vs. 12 DAI Control	7	98	105
	16 DAI RKN vs. 16 DAI Control	18	225	243
	20 DAI RKN vs. 20 DAI Control	62	71	133
	Total:	247 (30.5%)	562 (69.5%)	809
M120	4 DAI RKN vs. 4 DAI Control	4	66	70
	8 DAI RKN vs. 8 DAI Control	312	409	721
	12 DAI RKN vs. 12 DAI Control	95	82	177
	16 DAI RKN vs. 16 DAI Control	179	314	493
	20 DAI RKN vs. 20 DAI Control	65	136	201
	Total:	655 (39.4%)	1007 (60.6%)	1,662
**Grand Total**		**902 (36.5%)**	**1569 (63.5%)**	**2471**

^a^Interactions between C201 or M120 with southern root-knot nematode are characterized as compatible or incompatible, respectively. DAI refers to days after inoculation

#### DEGs during compatible interactions

**Fig. 2:**
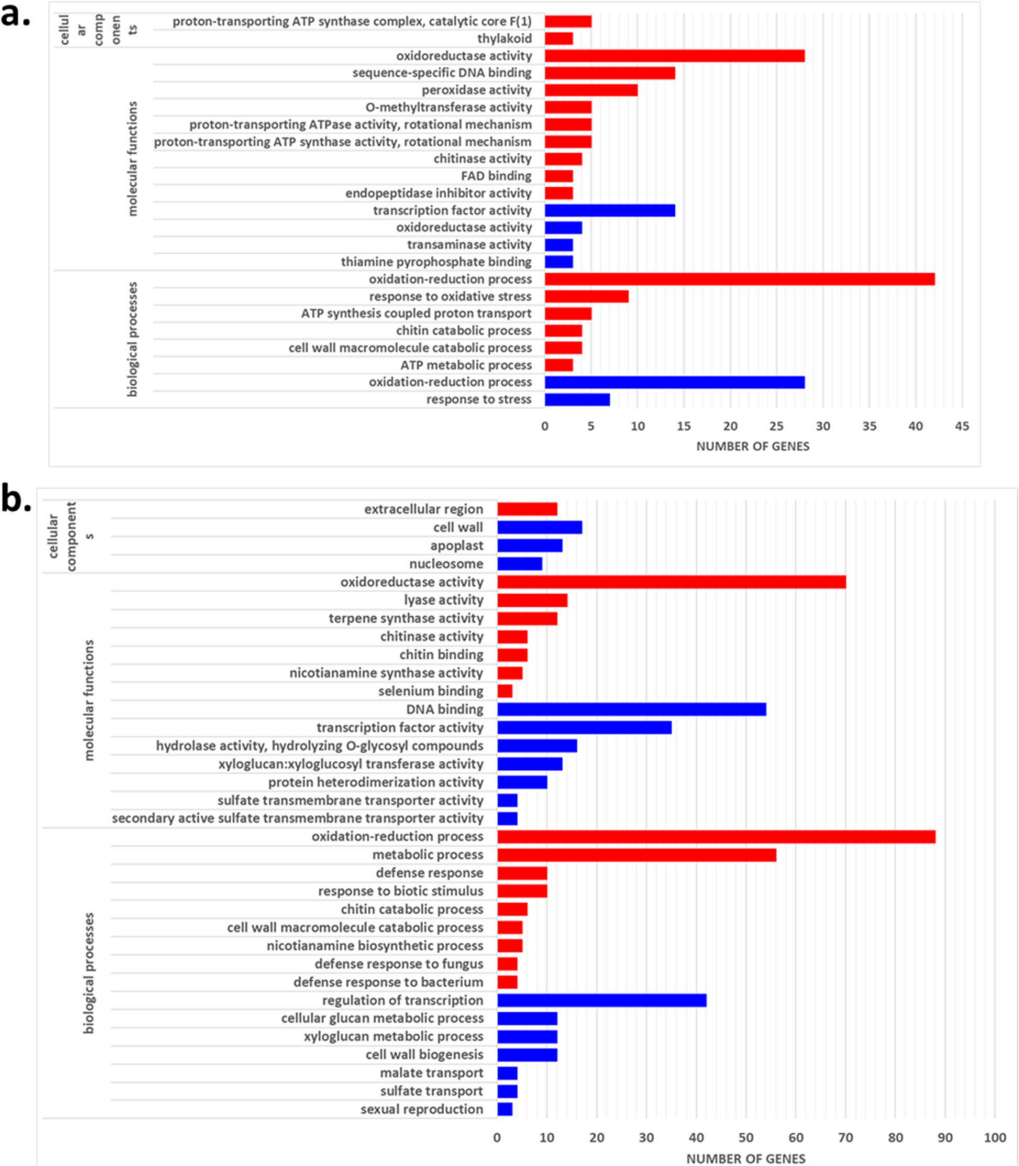
Enriched categories of DEGs during compatible and incompatible interactions. ‘Red’ bars correspond to upregulated and ‘blue’ downregulated categories of genes in **a**. C201 and **b**. M120

**Fig. 3:**
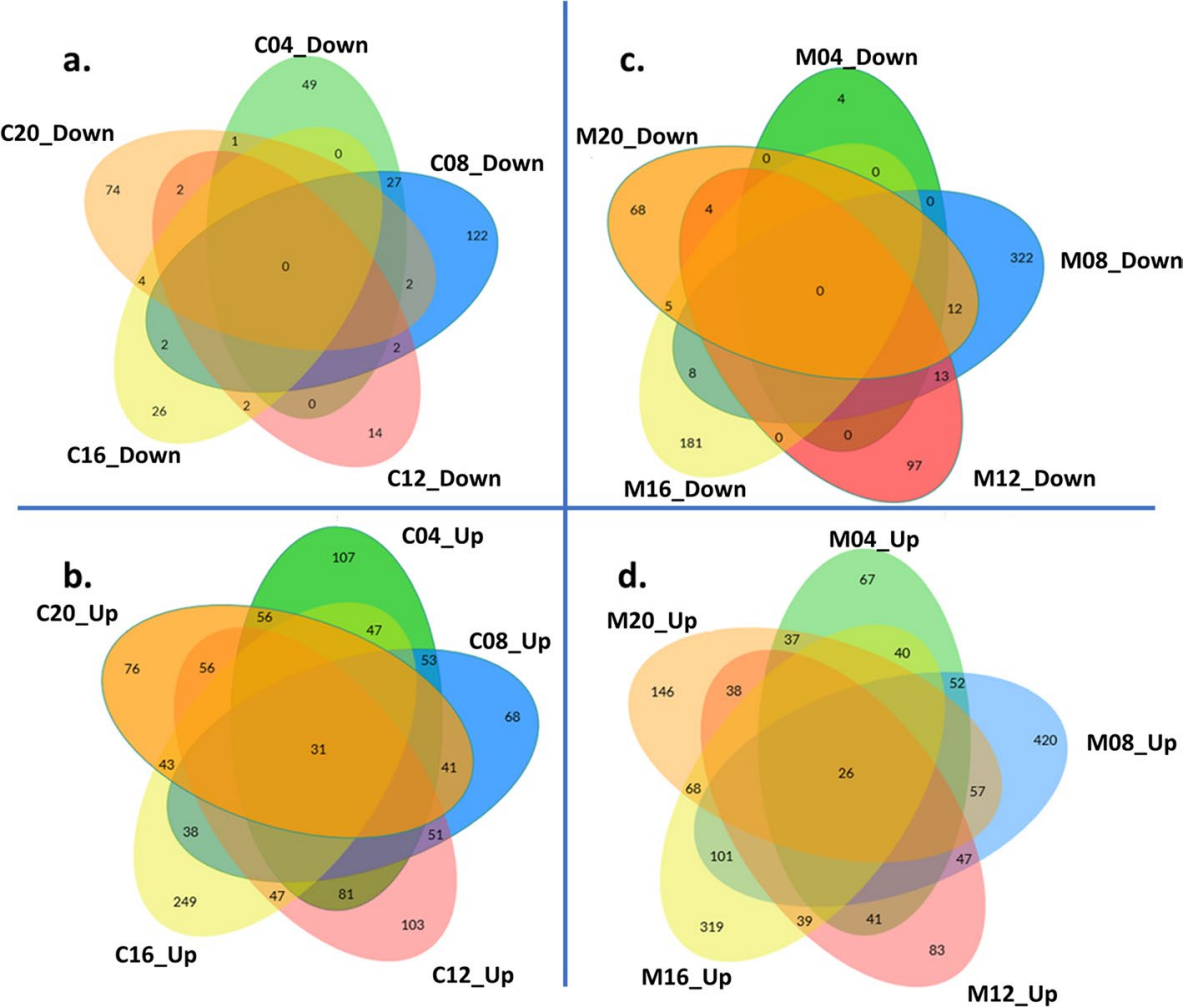
Venn diagrams of common DEGs during compatible and incompatible interactions at different DAI. Diagrams show **a**. downregulated genes in C201, **b**. upregulated genes in C201, **c**. downregulated genes in M120 and **d**. upregulated genes in M120, each at different days after inoculation (DAI). ‘C’ and ‘M’ correspond to C201 and M120, respectively. Numbers following ‘C’ and ‘M’ correspond to different DAI. ‘Up’ and ‘Down’ correspond to upregulated and downregulated. For DEGs common between two different DAI, significance threshold was kept at (FDR < 0.05) given that at least one of the occurrences is also significant at 4-log_2_ FC.

A total of 247 downregulated and 562 upregulated DEGs were significant during compatible interactions in the C201 genotype (Supplemental Table S1). Nearly half of the downregulated genes were observed at 8 DAI, whereas the highest number of upregulated genes (40%) were found at 16 DAI. Downregulated DEGs were enriched in gene ontology (GO) categories such as stress response and oxidation-reduction (Fig. 2a). A total of 27 downregulated genes were common between 4 and 8 DAI, indicating an early transcriptomic repression response during compatible interactions (Fig. 3a). In contrast, relatively few downregulated genes (1 to 4) were recurrent at other time points. Transcriptomic repression at 12 and 16 DAI was less dynamic than at 8 DAI, with only seven and 18 genes significantly downregulated, respectively. Conversely, 59% of upregulation events were recurrent across different time points (Fig. 3b; Supplemental Table S1). Enriched GO classes included biological processes such as oxidation-reduction, response to oxidative stress, and chitin catabolism (Fig. 2a).

#### DEGs during incompatible interactions

During incompatible interactions, 655 downregulated and 1,007 upregulated DEGs were significant (Supplemental Table S1). As observed in compatible interactions, nearly half of the downregulated genes occurred at 8 DAI, while the highest proportion of upregulated genes (~ 40%) were also detected at this time point. Approximately 41% of the DEGs were recurrent across at least one other sampling time point (Fig. 3c; Supplemental Table S1). At the earliest time point (4 DAI), only four genes were significantly downregulated, and no specific enrichments were observed at the final time point (20 DAI). Enriched categories for downregulated DEGs included regulation of transcription, cell wall biogenesis, and carbohydrate metabolic process, as well as associated processes like xyloglucan and cellular glucan metabolism and hydrolase activity of O-glycosyl compounds (Fig. 2b). Fewer downregulated genes were observed at 12 and 16 DAI (Supplemental Table S1). Among the upregulated genes, approximately 41% were recurrent across at least one other time point (Fig. 3d; Supplemental Table S1). Key enriched biological processes included metabolic processes like oxidation-reduction, nicotianamine biosynthesis, and chitin and cell wall macromolecule catabolism. Additionally, defense responses to biotic stimuli, including bacteria and fungi, were significantly enriched (Fig. 2b).

#### Common DEGs during compatible and incompatible interactions

A total of 0, 8, 4, 7, and 9 downregulated genes and 54, 52, 45, 100, and 37 upregulated genes were common between C201 and M120 at 4, 8, 12, 16 and 20 DAI, respectively (Supplemental Figure S2). No genes showed contrasting expression patterns (i.e., opposite directions) between the two genotypes at the same time point. While none of the common downregulated genes were recurrent across time points, several common upregulated genes were recurrent across different time points. For example, 16 DEGs, including DNA-directed RNA polymerase subunit beta, were upregulated in both genotypes throughout the infection. Similarly, two ATP synthase subunit alpha (*ATPA*) genes were recurrently upregulated. Gh_A03G1169 was upregulated at 4 and 12 DAI during compatible interactions and at all time points except 16 DAI during incompatible interactions. A second gene, Gh_D06G0518, was upregulated at all time points in both interactions except at 20 DAI during incompatible interactions.

#### Heat maps

**Fig. 4:**
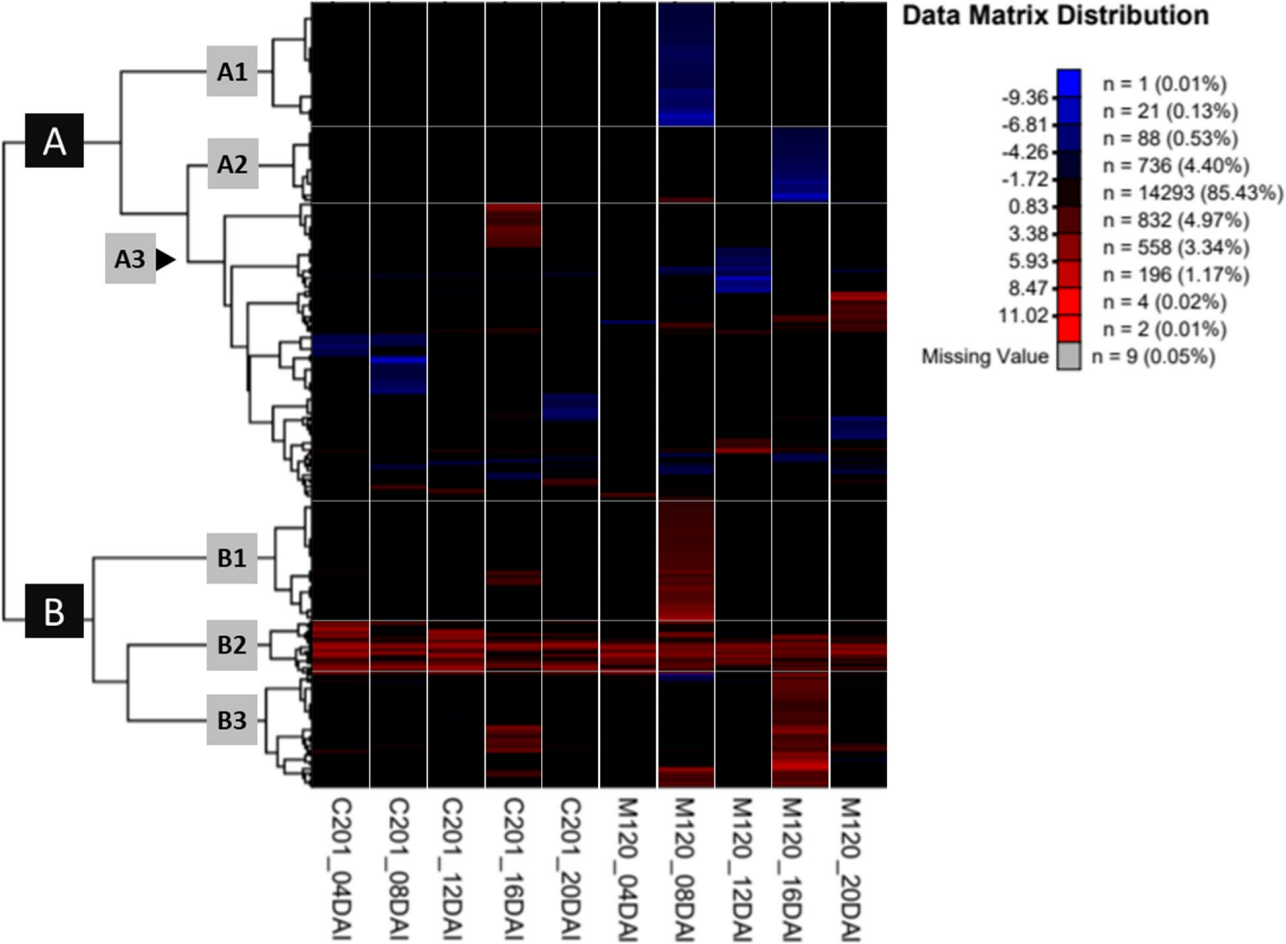
Heatmap clustering DEGs during compatible and incompatible interactions. The map shows clustering of DEGs at five different DAI (4, 8, 12, 16 and 20 DAI) in C201 (compatible) and M120 (incompatible). Upregulated DEGs identified with different shades of *Red* whereas downregulated are shown with different shades of *blue*. *Black* shade indicates that the genes do not display significant differential expressions.

Hierarchical clustering of significant DEGs revealed two primary gene clusters (Group A and B), each further divided into three sub-clusters (Fig. 4). Sub-clusters A1 and B1 contained down- and upregulated genes at 8 DAI, whereas A2 and B3 contained those at 16 DAI, respectively – these four clusters were predominantly associated with incompatible interactions. Sub-cluster A3 contained both up- and downregulated genes involved in both types of interactions, whereas B2 was primarily composed of genes that were upregulated during both compatible and incompatible interactions.

### Differentially expressed genes at the QTL regions

The physical locations of the QTL regions on chromosome 11 (designated as ‘A11’ in subgenome ‘A’) and chromosome 14 (designated as ‘D02’ in subgenome ‘D’) were determined by BLAST homology search against the *G. hirsutum* genome [28]. This search utilized clone sequences of SSR markers CIR316 and CIR069 spanning *qMi-C11*, and BNL3644 and NAU5467 spanning *qMi-C14* [11, 14]. Additionally, we included chromosome D11, the homeolog of A11, and chromosome A02, the homeolog of D02, to catalog DEGs in the QTL homoeologs. For genotype-specific contrasts at different DAIs, a liberal threshold (FDR < 0.05; log2 FC ≤ −1 or ≥ + 1) was applied to declare DEGs. In total, 47 differentially expressed transcripts corresponding to 38 genes within the two QTLs and their homoeologous regions were identified (Table 2).

**Table 2:** List of DEGs at the QTL regions

Chr.^*a*^	Gene id	Gene^*b*^	Description	Geno^*c*^	DAI	log_2_FC^*d*^
A11	Gh_A11G2836	*RPPL1*	Putative disease resistance RPP13-like protein 1	M120	8	2.02
	Gh_A11G2856	*XYL1*	Alpha-xylosidase 1	M120	8	-1.17
	Gh_A11G2865	*IQM3*	IQ domain-containing protein	M120	16	-1.92
D11	Gh_D11G3190	*RGA4*	Putative disease resistance protein	M120	8	1.63
	Gh_D11G3231	*XYL1*	Alpha-xylosidase 1	M120	8	-1.68
	Gh_D11G3245	*na*	*na*	M120	16	-2.17
	Gh_D11G3247	*IQM3*	IQ domain-containing protein	M120	8	1.65
A02	Gh_A02G0134	*na*	*na*	M120	12	1.94
	Gh_A02G0154	*At1g14780*	MACPF domain-containing protein	M120	8	1.32
	Gh_A02G0165	*CYS2*	Cysteine proteinase inhibitor 2	M120	8	-1.88
				M120	20	-2.24
	Gh_A02G0172	*CBDAS2*	Cannabidiolic acid synthase-like 1	M120	8	4.41
				M120	16	4.50
	Gh_A02G0174	*TMK4*	Receptor-like kinase	M120	8	-1.30
	Gh_A02G0178	*SBP2*	Selenium-binding protein 2	C201	16	2.67
				M120	16	5.12
	Gh_A02G0182	*HSFA5*	Heat stress transcription factor A-5	M120	8	1.07
	Gh_A02G0219	*na*	Cytochrome P450 CYP749A22	M120	8	1.47
	Gh_A02G0220	*na*	Cytochrome P450 CYP749A22	M120	8	1.17
	Gh_A02G0242	*na*	Glutamine synthetase leaf isozyme, chloroplastic	M120	8	-1.72
				C201	12	-1.67
	Gh_A02G0276	*na*	Endo-1,3	M120	8	1.05
D02	Gh_D02G0169	*na*	*na*	M120	12	1.09
	Gh_D02G0191	*At1g14780*	MACPF domain-containing protein	M120	8	1.34
	Gh_D02G0196	*WAKL8*	Wall-associated receptor kinase-like 8	M120	16	7.25
				M120	20	-7.51
	Gh_D02G0205	*BHLH62*	Transcription factor	C201	20	-1.18
	Gh_D02G0206	*CYS2*	Cysteine proteinase inhibitor 2	M120	20	-3.64
	Gh_D02G0213	*na*	Tetrahydrocannabinolic acid synthase	C201	16	2.08
	Gh_D02G0216	*CBDAS2*	Cannabidiolic acid synthase-like 1	M120	8	2.93
	Gh_D02G0218	*na*	*na*	C201	16	6.22
	Gh_D02G0220	*na*	Xyloglucan endotransglucosylase/hydrolase 2	C201	20	-1.61
				M120	20	-2.26
	Gh_D02G0241	*SBP2*	Selenium-binding protein 2	M120	16	2.33
	Gh_D02G0242	*SBP2*	Selenium-binding protein 2	M120	16	6.34
				M120	20	4.62
	Gh_D02G0257	*RLP12*	Receptor-like protein 12	C201	16	3.53
	Gh_D02G0259	*RLP12*	Receptor-like protein 12	M120	8	1.95
				M120	12	1.62
				M120	16	2.10
	Gh_D02G0289	*na*	Cytochrome P450 CYP749A22	M120	8	1.21
	Gh_D02G0291	*APL*	Myb family transcription factor	M120	8	1.34
	Gh_D02G0311	*na*	Glutamine synthetase leaf isozyme, chloroplastic	M120	8	-1.68
	Gh_D02G0328	*GSTU7*	Glutathione S-transferase U7	M120	8	2.01
	Gh_D02G0326	*GSTU7*	Glutathione S-transferase U7	M120	20	2.69
	Gh_D02G0327	*na*	*na*	M120	20	9.27
	Gh_D02G0329	*na*	Probable glutathione S-transferase	C201	16	-1.41

^a^Chromosome, ^b^*na* not available, ^c^C201 and M120 represent compatible and incompatible interactions, respectively, ^d^negative log_2_FC are down-regulated. DEGs significant at adjusted *p*-value of 0.05 and log_2_FC > 1

#### DEGs at qMi-C11 QTL region

Three DEGs were identified in the A11 QTL region during incompatible interactions: ‘putative disease resistance RPP13-like protein 1’ (*RPPL1*: Gh_A11G2836), ‘alpha-xylosidase 1’ (*XYL1*: Gh_A11G2856), and ‘IQ domain-containing protein’ (*IQM3*: Gh_A11G2865). *RPPL1* was upregulated at 8 DAI, whereas *XYL1* and *IQM3* were downregulated at 8 and 16 DAI, respectively. Notably, the QTL homoeolog in D11 contained a ‘putative disease resistance protein RGA4’ (Gh_D11G3190), which was upregulated at 8 DAI, whereas the homoeologs of *XYL1* and *IQM3* (Gh_D11G3231 and Gh_D11G3247) were significantly downregulated at 8 DAI. No significant DEGs were found in the *qMi-C11* QTL region during compatible interactions. qPCR indicated the upregulation of Gh_A11G2836 at 8 DAI in both compatible and incompatible interactions (Supplemental Figure S3c).

#### DEGs at qMi-C14 QTL region

**Fig. 5:**
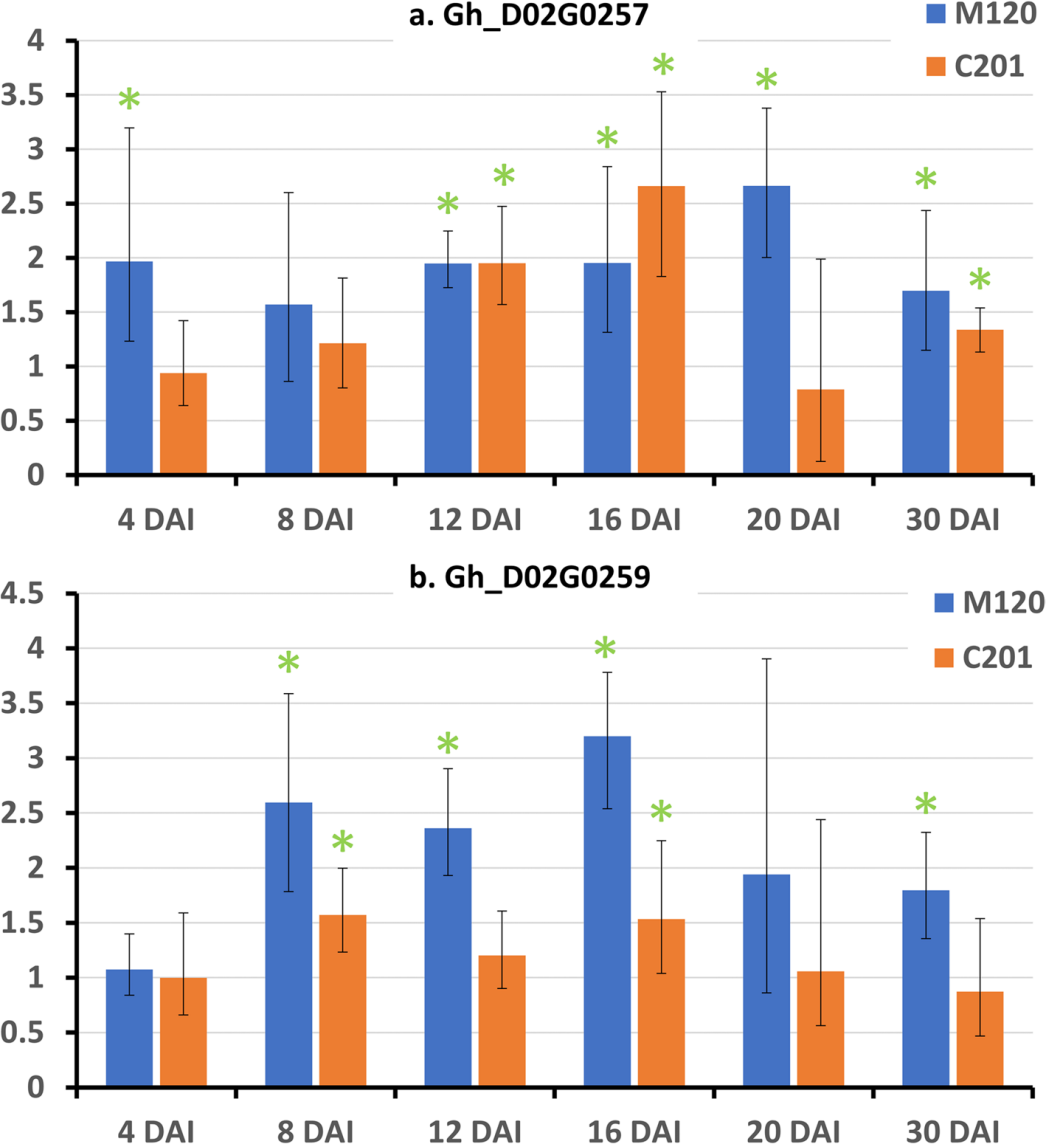
Quantitative real-time PCR (qRT-PCR) of q*Mi-C14* candidate genes a. Gh_D02G0257 and Gh_D02G0259. Charts show mean fold-change (with standard error bars) in expression at six different time points after RKN infection. Asterisks mark significant upregulation in expression in inoculated samples compared to non-inoculated plants and as determined by t-test of ΔCt values (*P* ≤ 0.05) using two biological replicates.

Among the 15 DEGs identified in the *qMi-C14* QTL region, three showed differential expression at multiple time points. Notably, ‘receptor-like protein 12’ (Gh_D02G0259) was upregulated at three consecutive time points—8, 12 and 16 DAI—during incompatible interactions. Additionally, six genes from D02 and its homoeolog (A02) were differentially expressed, such as ‘MACPF domain-containing protein’ (*At1g14780*: Gh_A02G0154 and Gh_D02G0191), ‘cysteine proteinase inhibitor 2’ (*CYS2*: Gh_A02G0165, Gh_D02G0206), ‘cannabidiolic acid synthase-like 1’ (*CBDAS2*: Gh_A02G0172, Gh_D02G0216), ‘cytochrome P450 *CYP749A22*’ (Gh_A02G0220, Gh_D02G0289), ‘selenium-binding protein 2’ (*SBP2*: Gh_A02G0178, Gh_D02G0241), and ‘glutamine synthetase leaf isozyme, chloroplastic’ (Gh_A02G0242, Gh_D02G0311). Both *SBP2* (Gh_A02G0178) and ‘glutamine synthetase’ gene were also differentially expressed during compatible interactions. Additionally, four genes, including ‘receptor-like protein 12’ (*RLP12*: Gh_D02G0257), showed differential expression during compatible interactions. qPCR analysis of the two putative ‘receptor-like protein 12’ candidate genes, Gh_D02G0257 (Fig. 5a) and Gh_D02G0259 (Fig. 5b), confirmed their significant overexpression in response to nematode parasitism. Gh_D02G0259 was upregulated beginning at 8 DAI and remained so through 16 DAI during incompatible interactions, further confirming the RNA-seq findings.

### Specific categories of nematode-responsive genes

#### Stress- and defense-responsive genes

The CottonFGD search function identified defense-related genes associated with the JA and SA pathways, resistance gene analogs (RGAs), pathogenicity-related (PR) genes, and ethylene biosynthesis. Genes involved in plant defense responses, including those in the alpha-linolenic acid metabolism (JA pathway), RGAs and PR genes, were differentially regulated in both compatible and incompatible interactions (Supplemental Table S2). In the alpha-linolenic acid pathway, which is a precursor of JA, two and four genes were repressed, while four and 14 genes were overexpressed during compatible and incompatible interactions, respectively (Supplemental Figure S4).

Further, Arabidopsis orthologs were identified for the significant DEGs found during *G. hirsutum*-*M. incognita* interactions. The NEMATIC database was used to identify common interactions between Arabidopsis and cotton in response to RKN (Supplemental Table S3). Focusing on nematode-responsive genes in the ‘stress’ category led to the identification of 25 common DEGs in the stress category during RKN-host interactions (Supplemental Table S4). Of these, six genes were downregulated during compatible interactions in both Arabidopsis and cotton with RKN.

#### Cell wall biogenesis and remodeling

Nematode-responsive DEGs involved in cell wall biogenesis, cell wall macromolecule catabolism, cell wall modification, and cell wall organization were identified (Supplemental Table S5). Incompatible interactions exhibited greater diversity in GO categories and a higher number of DEGs in response to nematode parasitism than in compatible interactions. These DEGs were categorized into four major molecular functions: xyloglucan:xyloglucosyl transferase activity, chitinase activity, pectinesterase activity, and expansin enzyme family (Supplemental Figure S5a-d). For instance, all five genes encoding endochitinases were upregulated during compatible interaction at 16 DAI, with fold changes ranging from 2.6 to 20.2. While three of these genes were also upregulated during incompatible interactions, five other endochitinases were specific to incompatible interactions. The highest fold change was observed for *CHIA1* (FC = 54.7). Additionally, 17 DE events corresponding to seven members of ‘fasciclin-like arabinogalactan proteins’ were downregulated at 8 and/or 16 DAI during incompatible interactions, whereas only one gene was downregulated at 8 DAI during compatible interactions. Furthermore, several DEGs in the phenylpropanoid biosynthetic pathway indicated changes in cellular biogenesis in response to nematode infection (Supplemental Figure S6a-b).

#### Cell growth and development

Several genes involved in cell proliferation were differentially regulated in response to nematode parasitism (Supplemental Table S6). For example, during incompatible interactions, ‘phytosulfokine 3’ (*PKS3*) growth factors, which facilitate the positive regulation of plant cell proliferation, were upregulated at 8 and 16 DAI, whereas two putative *PSK6* homoeolog genes were downregulated at 12 DAI. Additionally, three types of ‘cyclin-dependent protein serine/threonine kinase regulators’ (*CYCA*, *CYCB* and *CYCD*), along with two ‘G2/mitotic-specific cyclin S13-6’ genes, which regulate the cell cycle, were downregulated during incompatible interactions. Identical expression patterns were observed for two classes of ‘COBRA-like proteins’ (*COBL1* and *COBL7*), which regulate cell expansion and cellulose crystallinity. Specifically, two sets of homoeologous *COBL7* and *COBL1* genes were downregulated at 8 DAI and 20 DAI, whereas *COBL4* was downregulated at 16 DAI.

#### Transcription factors

**Fig. 6:**
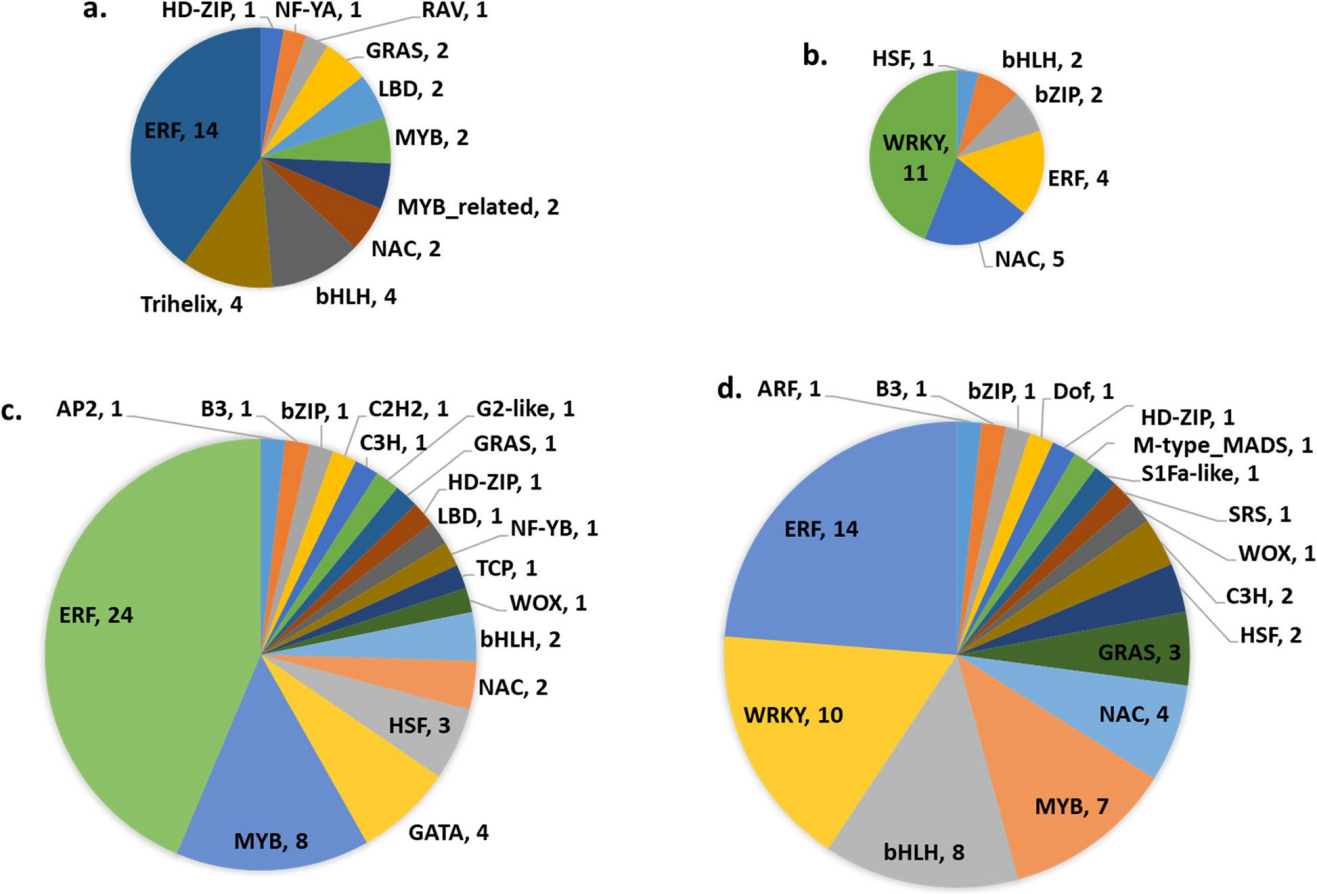
Distribution of *G. hirsutum* transcription factors (TF) families during RKN infection and development. Downregulated (**a** and **c**) and upregulated (**b** and **d**) TF families during compatible (**a** and **b**) and incompatible (**c** and **d**) interactions. Corresponding number of TF genes are provided following TF family identifiers (after *comma*)

Several transcription factor (TF) families altered expression patterns following RKN infection, likely driving widespread transcriptional changes. A total of 174 TFs across 29 families displayed differential expression in response to RKN infection (Fig. 6; Supplemental Table S7). During compatible interactions, 11 TF families were downregulated, and six were upregulated, whereas 18 and 17 families were differentially expressed during incompatible interactions. Among these, *WRKY* was one of the most abundant and exclusively overexpressed TF families of both interaction types. Similarly, ‘trihelix’ was repressed only in compatible interactions, while ‘GATA’ was downregulated only in incompatible interactions. In addition to these TF families, the expression of key genes involved in transcriptional regulation, such as ‘DNA-directed RNA polymerase’, was also altered. Notably, two to four ‘DNA-directed RNA polymerase’ genes (*rpoB*, *rpoB1* and *rpoB2*) were upregulated with an average fold change of 22 to 26 (average log_2_ FC > 4) across all sampling time points in both interaction types, supporting enhanced transcriptional activity in response to RKN infection.

#### Carbon and energy metabolism

A wide array of genes involved in glycolysis/gluconeogenesis, amino sugar and nucleotide sugar, and starch and sucrose synthesis pathways were differentially expressed in response to nematode parasitism during both compatible and incompatible interactions (Supplemental Table S8; Supplemental Figure S7-S10). For example, three genes corresponding to ‘basic vacuolar isoform of glucan endo-1,3-beta-glucosidase’ were upregulated (log_2_FC range from 4.1 to 5.3), whereas several plasma membrane-based members were moderately downregulated during incompatible interactions at 8 DAI. These genes were subsequently upregulated in both interaction types. Similarly, genes encoding ‘UDP-glucuronate 4-epimerase’ were downregulated in compatible interactions (two genes at 8 DAI) and in incompatible interactions (one gene at 16 DAI). Additionally, two ‘UDP-glucose 4-epimerase genes’ were upregulated during incompatible interactions at 16 DAI.

During compatible interactions, all DEGs associated with the glycolysis/gluconeogenesis pathway were downregulated at 4 and 8 DAI. For example, homoeologous copies of ‘alcohol dehydrogenase class-P’ (*ADH1*) and ‘pyruvate decarboxylase 1’ (*PDC1*) were downregulated at 4 and 8 DAI, whereas those of ‘ATP-dependent 8-phosphofructokinase 3’ (*PSK3*) were downregulated at 8 DAI. During incompatible interactions, *ADH1* was downregulated at 8 DAI but upregulated at 16 and 20 DAI. Additionally, a set of homoeologous *PSK3* genes was upregulated at 8 DAI during incompatible interactions. Similarly, ‘cytosolic fructose-1,6-bisphosphatase’, a key enzyme in the sucrose biosynthetic pathway, was upregulated at both 8 and 16 DAI.

## Discussion

Differential gene expression analysis using global transcriptome data is an effective approach to understand the regulatory networks of genes and pathways involved in plant host-pathogen interactions. Based on histopathological evidence, the current working hypothesis suggests that resistance in the Auburn 623 RNR source is conferred by two major genes with different mechanisms of resistance. The first mechanism occurs soon after infection (8–12 DAI), preventing juvenile nematodes from developing functional feeding sites, while the second mechanism occurs later (14–20 DAI), impeding the development of nematodes into egg-laying females [6–10]. Mechanisms of RKN resistance are dynamic biological processes, with nematode suppression occurring at multiple stages of the life cycle. Prior RNA-seq experiments, which were limited to a single time point (10 DAI) [27] or two time points (12 and 30 DAI) [26], provided important insights but were insufficient to fully capture the dynamics of RKN resistance. Therefore, the current study conducted a more detailed time-course RNA-seq experiment, constructing and sequencing 44 libraries across five time points (4, 8, 12, 16 and 20 DAI) from RKN-treated and untreated (controls) samples of C201 (susceptible) and M120 (resistant) genotypes. The DEGs identified between genotypes with and without nematode infections, as well as key candidate genes in the QTL regions, provide a more comprehensive view of the molecular events underlying RKN resistance in cotton.

###  Massive transcriptional reprogramming of defense response underlies cotton-RKN interactions

A dynamic transcriptional response distinguishes incompatible from compatible interactions. At 4 DAI, more DEGs were detected in compatible interactions (Table 1), supporting the idea that the gene types, rather than expression level, influence nematode survival [30]. From 8 DAI onward, incompatible interactions displayed stronger transcriptomic responses, both genome-wide and at specific QTL regions, despite higher nematode loads in C201 (Supplemental Table S9). The near-immunity in M120 appears to be driven by enhanced basal defense gene expression and selective activation of R genes—hallmarks of RKN resistance in several plant species, including tobacco [31, 32] and alfalfa [33]. In contrast, susceptibility involves active suppression of host defenses during nematode parasitism [29].

Resistance in M120 aligns with the zig-zag model of the plant immune system [34], involving two defense layers: nematode-associated molecular patterns (NAMPs)-triggered immunity (PTI) and effector-triggered immunity (ETI). PTI, associated with pathogenesis-related (PR) genes, is initiated by NAMP recognition via pattern recognition receptors (PRRs), triggering downstream cellular signaling cascades, including the activation of ‘mitogen-activated protein kinases’ (MAPKs), the production of reactive oxygen species (ROS), and the initiation of JA or SA pathways [35]. We detected 37 *Mi* genes (mapped to the *M. incognita* genome) encoding effector proteins (Supplemental Table S9) and several *Gh* genes encoding ‘receptor-like kinases’ (RLKs), receptor-like proteins’ (RLPs) and ‘wall-associated receptor kinases’ (WAKs) that were differentially expressed in RKN-treated samples. Six PR genes were differentially regulated during incompatible interactions, whereas only two were upregulated in compatible interactions. Furthermore, homoeologous RLK genes (*At1g72540*: Gh_A05G1164 and Gh_D05G1341) were downregulated in compatible interactions at 4 and 8 DAI, consistent with the general downregulation of PRR-encoding genes in susceptible hosts, as observed in potato [22].

One of the early PTI and/or ETI cellular responses involves the rapid generation of reactive oxygen species (ROS), which activates defense genes and reinforces cell wall [36]. In this study, several key ROS-associated genes were differentially expressed. For example, three different ‘peroxidases’ were downregulated at 8 DAI, whereas five ‘peroxidase’ genes and two ‘respiratory burst oxidases’ (*RBOHA*) were upregulated at 16 DAI during compatible interaction. This suggests nematodes suppress ROS-mediated defense during early infection, mirroring observations in potato [22]. Furthermore, the early (8 DAI) downregulation of ROS-scavenging genes coincides with histopathological evidence of localized HR during incompatible interactions [6], suggesting that while HR is inhibited in C201, it remains active in M120, though it does not involve classic HR-associated cell death.

’Patatin-like proteins’ (*PLP*s) are key mediators of HR in various host-pathogens interactions [37–39]. In this study, two *PLP1* and four *PLP2* genes were upregulated at 8 DAI during incompatible interaction (average log_2_FC = 4.85), potentially enabling limited HR, as suggested by histopathological studies in cotton [6]. Finally, the early induction of ‘dirigent’ genes in both this study and de Deus Barbosa et al. [30] suggests that during incompatible interactions, the endodermal barrier system actively impedes nematode migration toward the vascular tissues [40]. This barrier, absent in susceptible lines, may contribute to the higher success rate of nematodes in establishing feeding sites in C201.

### Several TF families show differential expression in response to *M. incognita* infection

The significant upregulation of the transcription catalysts (*rpoB*, *rpoB1* and *rpoB2*) during cotton-RKN interactions supports widespread transcriptional reprogramming in both compatible and incompatible interactions. Transcription factors (TFs), such as *WRKY* genes, play a crucial role in adaptive biotic stress responses. While *WRKY* genes were upregulated in both compatible and incompatible interaction types, they exhibited earlier induction, greater diversity, and in certain cases, a higher degree of upregulation during incompatible interactions. Notably, among the WRKY genes, the *SlyWRKY75* gene, a JA-pathway regulator [41], showed a striking 17- and 86-fold increase at 8 and 16 DAI, respectively, in RKN-treated M120, indicating a strong JA response. Additionally, six *WRKY70* genes were upregulated during compatible interactions, consistent with their role in mediating crosstalk between SA and ET/JA pathways [42], as observed in soybean- *M. incognita interactions* [43].

Ethylene response factors (ERF) are closely associated with hormonal signal transduction pathways that are involved in plant defense responses. In this study, the predominant downregulation of ERFs during compatible interactions suggests a suppressed ethylene response. However, multiple ERFs were differentially regulated during incompatible interactions, reflecting their complex role in RKN-cotton interactions, although reports of their functions remain inconclusive [44].

*MYB* TFs, known to mediate phytohormones defense signaling [45], were mostly downregulated in compatible interactions but displayed mixed regulation in incompatible interactions. Two *MYB108* genes were downregulated in compatible interactions, while four were upregulated in incompatible interactions, indicating enhanced downstream defense signaling responses. Similarly, three NAC TFs, which regulate cell wall remodeling in response to RKN infection [19] (Gh_A03G0024, Gh_A08G1428 and Gh_D03G1627), were significantly upregulated in both compatible and incompatible interactions but showed sustained overexpression in incompatible interactions at later stages.

### During cotton-RKN interactions, hormone-modulated responses abound

Phytohormones regulate plant defense by triggering defense molecules such as phenylpropanoids, phytoalexins, and pathogenicity-like proteins. Auxin-related genes exhibit differential expression during plant-nematode interactions [46, 47]. Indole-3-acetic acid (IAA), the primary natural form of auxin, is synthesized from tryptophan (Trp) via a two-step pathway: Trp is first converted to indole-3-pyruvate (IPA) by the TAA family aminotransferases, then IPA is converted to IAA by the YUC family flavin monooxygenases [48]. In this study, a TAA gene (*TAR4*) was upregulated at 16 DAI in both interactions, while *YUC10* genes (Gh_A08G1012 and Gh_D08G1285) were upregulated only in incompatible interaction at 8 DAI. Additionally, *NGA4*, a TF potentially linked to YUC overexpression [49], was upregulated at 16 DAI during incompatible interactions. Similarly, auxin-response factors *ARF5* and *ARF6* were overexpressed between 8 and 16 DAI in incompatible interactions, while *ARF7* and *ARF19* were upregulated only during compatible interactions at 20 DAI.

Nematodes also influence hormonal pathways, linked to root apical meristem maintenance, lateral root formation, and nematode feeding site development. Several SAUR and auxin-responsive genes were downregulated in compatible interactions, with mixed regulation in incompatible interactions. For example, *SAUR41* homologs (Gh_A10G0478 and Gh_D10G0502), involved in Arabidopsis cell expansion and root meristem patterning [50], were downregulated at 4 and 8 DAI in compatible interactions. Similarly, three LBD genes, which encode LOG domain-containing proteins associated with auxin-regulated root formation [51], were downregulated in response to nematode parasitism.

Genes involved in Jasmonic acid (JA) and Salicylic acid (SA) biosynthesis, key pathways in host resistance, were differentially expressed, particularly at 8 and 16 DAI. For instance, two genes encoding allene oxide synthase (*CYP74A*), a key enzyme in JA biosynthesis, were strongly upregulated (log_2_FC > 4.6) in incompatible interactions at 8 and 16 DAI. Other key genes of JA biosynthesis pathway were also upregulated, including *AOC4* (allene cyclase) and *LOX1.5* (lipoxygenase) [52] at 16 DAI, as well as several *ACX* (Acyl-coenzyme A oxidase) genes at 8 DAI. Similarly, the *PR-1* gene, a key SA-regulated defense gene [53], showed strong upregulation (FC = 32.6) in the incompatible interaction at 8 DAI. Additionally, several *PAL* genes, integral to phenylalanine-mediated SA production, were upregulated in both interactions, which is consistent with the response of *C. metuliferus* to RKN [20].

Hydrolases contribute to plant resistance to fungus, viruses, and plant-parasitic nematodes like *Heterodera rostochiensis * [54–56]. These enzymes, upregulated in incompatible interactions in cotton [30] and tobacco [32], are associated with hypersensitive responses (HR), which involve toxin accumulation, calcium flux, and cell death [34]. Such responses may deprive nematodes of nutrients, leading to giant cell collapse [20, 24]. In this study, three *HGN1* genes (Glucan endo-1,3-beta-glucosidase, basic vacuolar isoform), which activate toxin precursors, were strongly upregulated (average FC = 27.79) during incompatible interaction at 8 DAI, with one gene also upregulated at 16 DAI.

Ethylene-related genes, known to be induced in nematode feeding sites [57], also enhance systemic expression of basic chitinases involved in nematodes’ defense [58, 59]. In this study, endochitinases were mostly upregulated in both interactions, but incompatible interactions showed earlier induction, greater diversity, and higher upregulation. For instance, *endochitinase 1* expression had an average FC of 14.6 in compatible interactions at 16 DAI, but 54.7 and 27.4 in incompatible interactions at 8 and 16 DAI, respectively, aligning with soybean responses [60].

### DEGs involved in metabolic/transport activities and giant cell formation

Nematodes exploit host systems in compatible interactions to divert nutrients from the giant cells to their feeding sites. During these interactions, six *aminotransferases* were downregulated and three were upregulated, indicating reduced catabolism and increased allocation of amino acids to nematodes. Conversely, in incompatible interactions, the trend was reversed, with six aminotransferases upregulated and two downregulated, suggesting enhanced catabolism and restricted nutrient flow to nematodes. Also, genes linked to oligopeptide transport were mostly upregulated at 8 DAI (five upregulated and one downregulated) but showed repression at 16 DAI, reducing nutrient supply to giant cells. PSK (phytosulfokine) signaling, involved in nematode-induced redifferentiation in Arabidopsis [61], was repressed. In this study, four *PSK6* genes were downregulated in compatible interactions, and two in incompatible interactions. Gh_A01G1788 was recurrently downregulated at 12 and 16 DAI in both interactions and at 20 DAI in incompatible interactions. A homoeologous *PSK6* gene pair (Gh_A01G1788 and Gh_D01G2029) was also downregulated at 12 DAI across both interactions. Downregulation of ‘E2F-transcription factor’ gene (*E2FE*) and its modulator, the retinoblastoma-related (*RBR*) protein, at 8 DAI, along with repression of six cyclin-encoding genes, suggest that characteristic endoreduplication in giant cells was repressed during incompatible interactions [62]. The lower galling index and smaller galls in resistant genotypes, compared to the larger galls in susceptible genotype, suggest that differential regulation of *PSK3*, *PSKR*, *RBR*, *E2F* and cyclin genes during early infection plays a crucial role, as observed in soybean [43].

Basal responses to nematode-secreted cell wall-degrading enzymes involve strengthening the cell wall barrier through the action of endochitinases, pectinesterases, expansins, fasciclin-like arabinogalactan proteins, and dirigent proteins. Many of these genes were differentially expressed here and are conserved, nematode-responsive orthogroups in resistant genotypes [23]. Further, giant cell formation requires altered expression of genes regulating cell wall remodeling. Four such gene families—xyloglucan:xyloglucosyl transferase, chitinase, pectinesterase, and expansin—showed notable changes in both interactions. Additionally, phenylpropanoid biosynthesis, essential for physical and biochemical defenses (e.g., lignin and suberin), is critical for pathogen resistance [63]. Ye et al. reported that timely and efficient activation of this pathway distinguishes incompatible from compatible interactions [20]. This study confirmed significant expression changes in phenylpropanoid-related genes, with incompatible interactions exhibiting earlier induction, greater diversity, and higher upregulation.

### DEGs at the QTL regions emerge as key RKN-resistance candidates

DEGs within the QTL intervals of *qMi-C11* and *qMi-C14* are of particular interest in this study because transcriptional changes observed during incompatible interactions are likely driven by one or more genes that interfere with nematode parasitism [7]. A total of 22 genes within these QTL regions were differentially expressed during incompatible interactions, compared to only six during compatible interactions.

Previous findings showed that nematode survival and reproduction were markedly reduced in resistant lines, with a lower proportion of nematodes progressing to J3 (third-stage juvenile) and J4 (fourth-stage juvenile) stages in M120 compared to C201 at 8 DAI (3.4% vs 11.9%) [7]. The delay in J2s reaching the central cylinder and becoming sedentary aligns with the first two sampling time points in this study (4 and 8 DAI), where the highest number of DEGs was detected at 8 DAI. This supports the idea that the *qMi-C11* QTL mediates M120-specific defense responses early in the infection cycle. During this phase, J2s in compatible interactions would have developed into J3s, and hypertrophied giant cells would already be evident [6, 7]. In contrast, in incompatible interactions, necrotic and dying cells in the central cylinder, indicative of HR, would be prominent [6].

The period between the second and third sampling time points (8 to 12 DAI) corresponds to the HR phase, ultimately leading to nematode feeding site degeneration by the fourth and fifth sampling time points (16 to 20 DAI). Interestingly, the second highest number of DEGs were observed at 16 DAI, suggesting that the *qMi-C14* QTL activates defense responses that disrupt nematode development in the later stages of the infection cycle during incompatible interactions. This disruption likely prevents RKNs from developing into mature females, thereby reducing their reproductive capacity [7]. While *qMi-C14* appears critical for defense responses at later stages, the precise mechanism by which it confers resistance remains unclear and warrants further investigation.

For the *qMi-C11* QTL on chromosome 11, our previous study identified a pair of colocalized putative disease resistance genes (RPP13-like protein 1: Gh_A11G2835 and Gh_A11G2836) that exhibited contrasting expression patterns in RKN-treated M120 and C201 genotypes [26]. While Gh_A11G2835 did not show differential expression in the current study, two other *RPPL1* genes (Gh_A11G2836 and Gh_A11G2838) were downregulated in RKN-treated M120 versus C201 contrasts at 12 and 20 DAI. However, qPCR did not validate the downregulation at these time points (Supplemental Figure S3c). Notably, Gh_A11G2836 was upregulated during incompatible interactions at 8 DAI in both RNA-seq and qPCR analyses, which suggests dynamic regulation in response to nematode pathogenicity.

Another candidate gene, ‘U-box domain-containing protein 21’ gene (*PUB21*: Gh_A11G3090), located within the *qMi-C11* QTL region, was previously reported to be upregulated in the resistant line at both early (12 DAI) and late (30 DAI) infection stages [26]. Interestingly, several E3 ubiquitin ligases, including *PUB21* orthologs in Arabidopsis, are known to respond to chitin, a well-established plant defense elicitor in fungal cell walls and the exoskeletons of insects and nematodes [64]. While RNA-seq analysis in the current study did not identify Gh_A11G3090 as differentially expressed, qPCR indicated its upregulation at four and five different time points during compatible and incompatible interactions, respectively (Supplemental Figure S3d). The inconsistencies in DEGs identified within the *qMi-C11* region across RNA-seq studies or between RNA-seq and qPCR approaches may reflect subtle expression changes that initiate downstream resistance responses or suggest that post-transcriptional modifications are involved in *qMi-C11*-mediated resistance. Nevertheless, Gh_A11G2836 and Gh_A11G3090 remain the putative candidate genes within the *qMi-C11* QTL region.

In a recent study, Ojeda-Rivera et al. employed a single sampling time point (23-day-old seedlings) for their comparative transcriptomic analysis. They concluded that the basal expression of candidate genes might be constitutively higher in ‘Acala NemX’, potentially conferring resistance without imposing a fitness penalty [27]. Notably, higher basal expression levels were observed in two homologs of an Arabidopsis NBS-LRR R protein gene (AT5G36930), namely *Gohir.A11G297600* (log_2_FC = 1.28) and *Gohir.D11G312800* (log_2_FC = 1.99), in mock-treated libraries of ‘Acala NemX’ compared to ‘Acala SJ2’. As such, these genes were proposed as the most promising candidates for RKN-resistance in ‘Acala NemX’, particularly as they are located within the *rkn1* QTL region [27]. In the current study, these genes did not show differential expression in any of the contrasts examined, whether within genotypes (mock vs. treated) or between genotypes (mock vs. mock or treated vs. treated) across all time points. Furthermore, there were no shared DEGs between the two studies within the *qMi-C11* QTL region. This disparity is not unexpected, as the resistance source in this study, ‘Auburn 623 RNR’ (*qMi-C11*), and the ‘Acala NemX’-sourced *rkn1* QTL are likely different, even though the QTLs are mapped to the same chromosome region but in adjacent, yet distinct, marker-intervals. Further, resistance in ‘Acala NemX’ is conferred by a recessive gene, whereas in ‘Auburn 623 RNR’, it is conferred by dominant gene(s) [12, 65], strongly indicating that they have unique modes of action [12, 27]. Collectively, these results support the hypothesis that *rkn1* and *qMi-C11* represent unique and independent QTLs.

For the *qMi-C14* QTL on chromosome 14, our prior research identified two *RLP12* genes (Gh_D02G0257 and Gh_D02G0259) as promising candidates based on both RNA-seq and qPCR analyses [15]. In this study, comparative contrasts of RKN-treated genotypes revealed differential expression of these genes. Specifically, Gh_D02G0257 was relatively overexpressed in the susceptible line at 12, 16 and 20 DAI, whereas Gh_D02G0259 was consistently overexpressed in the resistant line across all time points (Supplemental Table S10). Further, RNA-seq analysis showed that Gh_D06G0257 was upregulated during compatible interaction at 16 DAI, whereas Gh_D06G0259 was upregulated during incompatible interactions at 8, 12 and 16 DAI, with highest fold change (log_2_FC = 2.1) at 16 DAI. These timelines align closely with histopathological observations from da Silva et al., who reported a six-fold reduction in nematodes progressing to females and a tenfold decrease in egg production in the resistant genotype (M120) at 16 and 20 DAI, respectively [7]. These findings strongly suggest that *RLP12* genes within the chromosome 14 QTL region may act as plant receptors capable of detecting nematode parasitism and initiating signaling pathways that activate innate immune responses, thereby restricting RKN reproduction in the resistant genotype.

It is worth noting that neither this study nor our earlier study [26] showed significant upregulation of Gh_D02G0276, a gene previously proposed as the casual gene for the *qMi-C14* [16], in response to RKN infection (Supplemental Figure S3f). This gene was identified in MAGIC RIL lines derived from M-240 RNR, whereas our studies used M-120 RNR; however, both share Auburn 623 as their source of resistance. Similarly, Ojeda-Rivera et al. reported significant upregulation of two putative candidate genes on chromosome 14, *Gohir.D02G023200* (log_2_FC = 3.28) and *Gohir.D02G023300* (log_2_FC = 2.73), in the susceptible genotype ‘Acala SJ2’ in response to nematode infection, whereas their expressions did not differ significantly in the resistant genotypes [27]. Although ‘Acala NemX’ does not carry the *qMi-Chr14* QTL [66], its presumed source, ‘WMJJ’ [13, 14], also showed no differential expression of these genes at 10 DAI [27]. Interestingly, basal expression comparisons (control libraries) revealed a significant difference for *Gohir.D02G023200* (log_2_FC = 4.42) between ‘Acala NemX’ and ‘Acala SJ2’, leading researchers to propose that ‘Acala NemX’ employs constitutive overexpression of these genes, rather than infection-induced changes, to suppress nematode infection [27]. These discrepancies in gene expression, observed in response to RKN infection, may reflect genotypic differences between ‘WMJJ’ and ‘M-120 RNR’.

## Conclusion

Basal defense responses were activated both during compatible and incompatible interactions, although the responses were more pronounced in RKN resistant genotypes compared to the susceptible lines. In summary, nematode responsive genes related to defense pathways were often repressed during compatible interactions, whereas earlier induction, greater diversity, and a higher degree of upregulation of those genes were archetypal of incompatible interactions. A wide spectrum of disease resistance and putative resistance genes, pathogenesis-related genes, and genes corresponding to ligands and receptors were differentially expressed in response to nematode parasitism in *G. hirsutum*. These genes were mapped across the cotton genome and include potential candidate genes Gh_A11G3090 (*PUB21*) and Gh_A11G2836 (*RPPL1*) in chromosome 11 and Gh_D02G0257 (*RLP12*) and Gh_D02G0259 (*RLP12*) in chromosomes 14 QTL regions.

Comparative transcriptomics of a whole root system has limitations, as it may mask, dilute, and/or modify transcriptomic changes at localized interaction sites, such as specific cells or tissues [46, 67]. However, using controls at each time point and comparing near isogenic lines can justify this broader approach. Also, since nematode infection and development within plant roots are not synchronous, pooling root tissues over several days, as done by Shukla et al. [21], may better capture these dynamic interactions. Ongoing research on comparative transcriptomics of near-isogenic lines with QTLs on chromosomes 11 and 14 aims to validate these findings and identify candidate genes for future functional studies that could explain the ‘near immunity’ levels of resistance in M-120 RNR.

## Methods

### Plant material and greenhouse evaluation

Two Upland cotton genotypes, M-120 RNR (M120) and Coker 201 (C201), with contrasting responses to RKN parasitism, were used in this study. C201 is an obsolete cotton variety that is susceptible to *M. incognita*, while M120 is a near-isogenic line highly resistant to both galling and RKN egg production. It was developed by crossing Auburn 634 RNR with C201, followed by several backcrosses to C201, before being self-pollinated and publicly released as M-120 RNR [68]. Selfed progenies of these two publicly available germplasm lines are continually developed and maintained in our breeding program. The greenhouse experiments used in this study are described in the companion study [7]. Briefly, seeds of both genotypes were germinated in vermiculite. Fourteen days after germination, seedlings were individually transplanted into 10.6 cm x 10.6 cm x 12.4 cm pots filled with approximately 500 ml of steam-pasteurized soil (Tifton loamy sand). At transplant, half of the seedlings of each line were infected with 4,000 *M. incognita* J2s per plant, while the other half was left inoculated, serving as control. The entire root systems from inoculated and un-inoculated M120 and C201 seedlings were carefully removed from the potting-mix at 4, 8, 12, 16 and 20 days after inoculation (DAI), washed with deionized water, dried with sterilized paper towels and immediately frozen in liquid nitrogen. Root tissues of uninoculated seedlings at 0 DAI were also harvested as controls. Samples were stored at −80°C until RNA extraction. In total, 44 samples were collected, representing the resistant (M120) and susceptible genotype (C201), two treatments (RKN-treated vs. control), five time points (4, 8, 12, 16 and 20 DAI), and two biological replications for each condition. Phenotypic data from the companion study [7] corresponding to *M. incognita* counts at different developmental stages in the roots of C201 and M-120 RNR are presented in Supplemental Tables 10 and 11.

### Library construction and sequencing

Frozen root samples from C201 and M120, collected at six different time points, were individually ground with mortar and pestle in liquid nitrogen. Total RNA was then extracted using the Spectrum™ Plant Total RNA extraction kit (Sigma-Aldrich), following the manufacturer’s protocol. Library construction was performed by the Georgia Genomics and Bioinformatics Core at the University of Georgia, Athens, GA, using the Kapa Stranded RNA-seq kit (Roche), and sequencing was conducted on a NextSeq PE150 High Output flow cell platform. The RNA sequencing data from this study have been deposited in the NCBI database under BioProject ID PRJNA1062816 and SRA accessions SRX23150901 to SRX23150944.

### Transcript assembly and differential expression analyses

The quality of raw reads was checked using the FastQC program (https://www.bioinformatics.babraham.ac.uk/projects/fastqc/). Low-quality bases and adapter sequences from the paired reads were trimmed using the Trimmomatic v0.30 program [69] (http://www.usadellab.org/cms/?page=trimmomatic). Trimmed reads were then mapped to the *G. hirsutum* reference genome [28] using HISAT2 v2.1.0 (https://daehwankimlab.github.io/hisat2/) [70]. Sequence alignment files were input into the software Stringtie v1.3.3 to assemble potential transcripts (http://ccb.jhu.edu/software/stringtie/) [71]. A Python script was used to obtain gene-level raw counts from each library for differential gene expression analysis using the R package DESeq2 (https://bioconductor.org/packages/release/bioc/html/DESeq2.html) [72].

DESeq2 package, incorporated in SARTools [73], an R-based comprehensive differential expression (DE) analysis platform, was run with default parameters for RNA-seq data analysis. Adjusted *P*-value of ≤ 0.05, based on Benjamini-Hochberg procedure, was used to declare significant DE genes, while ‘cooksCutoff’ was used for outlier detection and ‘independentFiltering’ was used to remove low count genes. Contrasts corresponding to DE of genes within the genotypes (C201 or M120) under different conditions (RKN treatment vs. control) and time points (4, 8, 12, 16 and 20 DAI), as well as between the genotypes (M120 vs. C201) under identical conditions and time points, were also performed. The web-based Next-Generation Clustered Heat Map (NG-CHM) (https://www.ngchm.net/) was employed using Ward’s agglomerative hierarchical clustering and Euclidean metric to cluster significant DEGs and generate heat maps [74].

### Functional characterization, gene enrichment analysis, and real-time qPCR

Differentially expressed genes (DEGs) involved in plant defense response and disease resistance, in signaling (such as ligands, receptors, and transcription factors), or in cell wall and cellular functions were identified using keyword search tools available in the Cotton Functional Genomics Database CottonFGD (https://cottonfgd.net/) [75]. Gene ontology (GO) analysis, gene list enrichment, and KEGG pathway analyses were performed using the ‘Data Fetch and Enrichment’ tool in CottonFGD. Default parameters of *P* ≤ 0.0001 and ‘Minimum Gene Number’ of 3 were used to produce enrichment categories. Graphical pathways were obtained from KEGG Mapper’s Search&Color Pathway web tool (https://www.genome.jp/kegg/tool/map_pathway2.html). FunRich [76] and Genvenn (http://habtom.github.io/biojs-vis-genvenn/examples/index.html) were used for the graphical display of Venn diagrams. Expression patterns of common genes and orthologs of identified DEGs were further explored using the NEMATIC database [77], an Excel-based resource harboring several *A. thaliana*-RKN transcriptomic studies.

Details of primer design and RT-qPCR-based relative gene expression analysis (normalized against endogenous *GhACT4*) for putative candidate genes within the *qMi-C11* and q*Mi-C14* QTL regions have been previously reported [26]. Briefly, two biological replications of C201 and M120 from six different time points post-inoculation (including 30 DAI, which was not sequenced) and their respective controls were analyzed following Kumar et al. [26].

## Supplementary Information

Supplementary Material 1: Supplemental Table S1. Differentially expressed genes (DEGs) during compatible and incompatible interactions between *G. hirsutum*-*M. incognita* interactions.

Supplementary Material 2: Supplemental Table S2. Stress- and defense-related DEGs.

Supplementary Material 3: Supplemental Table S3. Significant DEGs identified during compatible and incompatible *G. hirsutum*-*M. incognita* interactions and their putative nematode-responsive homologous genes in Arabidopsis cataloged in NEMATIC.

Supplementary Material 4: Supplemental Table S4. Significant DEGs identified during compatible and incompatible *G. hirsutum-M. incognita* interactions and their putative nematode-responsive orthologous genes in Arabidopsis cataloged in 'stress' category in NEMATIC.

Supplementary Material 5: Supplemental Table S5. Cell wall biogenesis and remodeling related DEGs.

Supplementary Material 6: Supplemental Table S6. Cell growth and development-related DEGs.

Supplementary Material 7: Supplemental Table S7. Differentially expressed transcription factors (TFs).

Supplementary Material 8: Supplemental Table S8. DEGs related to carbon and energy metabolism.

Supplementary Material 9: Supplemental Table S9. Detailed information of annotated *M. incognita* genes identified in RNA-seq libraries and includes RNAi pathway proteins, carbohydrate active enzymes (CAZymes) and nematode effectors.

Supplementary Material 10: Supplemental Table S10. Differentially expressed genes (DEGs) in contrasts corresponding to compatible and incompatible interactions between *G. hirsutum*-*M. incognita* interactions.

Supplementary Material 11: Supplemental Figure S1. Distribution of mapped and unmapped paired-end sequences corresponding to two different genotypes (‘C’ C201 and ‘M’ M120), six different time points (‘0’, ‘4’, ‘8’, ‘12’, ‘16’ and ‘20’), and two different treatments (‘Cont’ control and ‘RKN’ treated with RKN).

Supplementary Material 12: Supplemental Figure S2. Number of common DEGs during compatible and incompatible interactions at different DAI.
Diagrams show a. Venn diagram of upregulated genes (‘red font’ are total numbers for the group), b. Venn diagram of downregulated genes, and c. frequencies and percentages of upregulated genes.

Supplementary Material 13: Supplemental Figure S3. Quantitative real-time PCR (qRT-PCR) of candidate root-knot nematode (RKN) resistance genes at six different infection time points corresponding to two different genotypes (‘C’ C201 and ‘M’ M120), six different time points (‘4’, ‘8’, ‘12’, ‘16’, ‘20’, and ’30’ days after infection, DAI), and two different treatments control and treated with RKN).
β- actin gene was used as reference gene (endogenous control) for normalizing the data. Charts show mean fold-change (with standard error bars) in relative expression of target genes against the endogenous control after RKN infection. *Green* asterisks mark significant upregulation, and *red* asterisks mark significant down regulation in expression in inoculated samples compared to non-inoculated plants and as determined by t-test of ΔCt values (*P* ≤ 0.05) using two biological replicates each with three technical replicates per sample.

Supplementary Material 14: Supplemental Figure S4. Expression profiles of the genes in alpha-linolenic acid pathway during compatible and incompatible interactions.
The heat map (a) shows DEGs from C201 (compatible) and M120 (incompatible) samples at five different time points (4, 8, 12, 16 and 20). Darker shades of *blue* and *red* bars indicate greater magnitude of down- or upregulation of DEGs, whereas *black* suggests indifferentiable expression. The pathway (b) shows upregulated (*red* highlighted) and downregulated (*blue* highlighted) genes during incompatible interaction at 8 DAI.

Supplementary Material 15: Supplemental Figure S5. Expression profiles of the genes encoding enzymes involved in cell wall biogenesis represented by xyloglucan endotransglucosylase/hydrolase protein (a), cell wall macromolecule catabolic process dominated by chitinase and endochitinases (b), cell wall modification represented by pectinestarase (c), and cell wall organization represented by expansins (d) during compatible and incompatible interactions between *G. hirsutum* and *M. incognita* at five different time points. Darker shades of *blue* and *red* bars indicate greater magnitude of down- or upregulation of DEGs, whereas *black* suggests indifferentiable expression.

Supplementary Material 16. Supplemental Figure S6. Expression profiles of the genes encoding enzymes involved in phenylpropanoid biosynthetic pathway during compatible and incompatible interactions between *G. hirsutum* and *M. incognita* at five different time points (a), and phenylpropanoid biosynthesis pathway at 8 DAI in the incompatible interaction (b). Heatmap: darker shades of *blue *and *red* bars indicate greater magnitude of down- or upregulation of DEGs, whereas black suggests indifferentiable expression. The pathway (b) shows upregulated (*red* highlighted) and downregulated (*blue* highlighted) genes during incompatible interaction at 8 DAI.

Supplementary Material 17: Supplemental Figure S7. Expression profiles of the genes encoding enzymes involved in amino sugar nucleosugar metabolism (a), glycolysis/gluconeogenesis (b), and starch and sucrose metabolism (c) during compatible and incompatible interactions between *G. hirsutum* and *M. incognita* at five different time points. Darker shades of *blue* and *red *bars indicate greater magnitude of down- or upregulation of DEGs, whereas *black*suggests indifferentiable expression.

Supplementary Material 18: Supplemental Figure S8. Expression profiles of the genes encoding enzymes in amino sugar and nucleotide sugar pathway at 8 (‘left’) and 16 (‘right’) DAI in an incompatible interaction between *G. hirsutum* and *M. incognita*. The pathways show upregulated (*red* highlighted) and downregulated (*blue* highlighted) genes.

Supplementary Material 19: Supplemental Figure S9. Expression profiles of the genes encoding enzymes in glycolysis/gluconeogenesis pathway at 8 DAI in a compatible interaction (‘left’) and 8 and 16 DAI (‘center’ and ‘right’) in an incompatible interaction between *G. hirsutum* and *M. incognita*. The pathways show upregulated (*red* highlighted) and downregulated (*blue* highlighted) genes.

Supplementary Material 20: Supplemental Figure S10. Expression profiles of the genes encoding enzymes in starch and sucrose metabolism pathway at 8 (‘left’) and 16 (‘right’) DAI in an incompatible interaction between *G. hirsutum* and *M. incognita*. The pathways show upregulated (*red* highlighted) and downregulated (*blue* highlighted) genes.

Supplementary Material 21: Supplemental Table S11. Number of *Meloidogyne incognita* (different developmental stages) in the roots of cotton near isolines that differ in *M. incognita* resistance genes.

Supplementary Material 22: Supplemental Table S12. Percentages of *Meloidogyne incognita* (different developmental stages) in the roots of cotton near isolines that differ in *M. incognita* resistance genes.

## Data Availability

The plant materials used in this study are publicly available resources. The raw RNA-seq data supporting the conclusions of this article are available through the National Center for Biotechnology Information database (https://www.ncbi.nlm.nih.gov/), under BioProject (I.D. PRJNA1062816) titled ‘Global transcriptome of Southern root-knot nematode resistant and susceptible Upland cotton genotypes.’ Direct link to datasets: http://www.ncbi.nlm.nih.gov/bioproject/1062816. Processed data are included within the manuscript or provided as Supplemental Files.
