# Supplementary figures and images for: Time-course RNA-seq analysis of upland cotton (Gossypium hirsutum L.) responses to Southern root-knot nematode (Meloidogyne incognita) during compatible and incompatible interactions
Source: BMC Genomics. 2025 Feb 24;26:183. doi: 10.1186/s12864-025-11339-w (PMC11849305; doi:10.1186/s12864-025-11339-w)

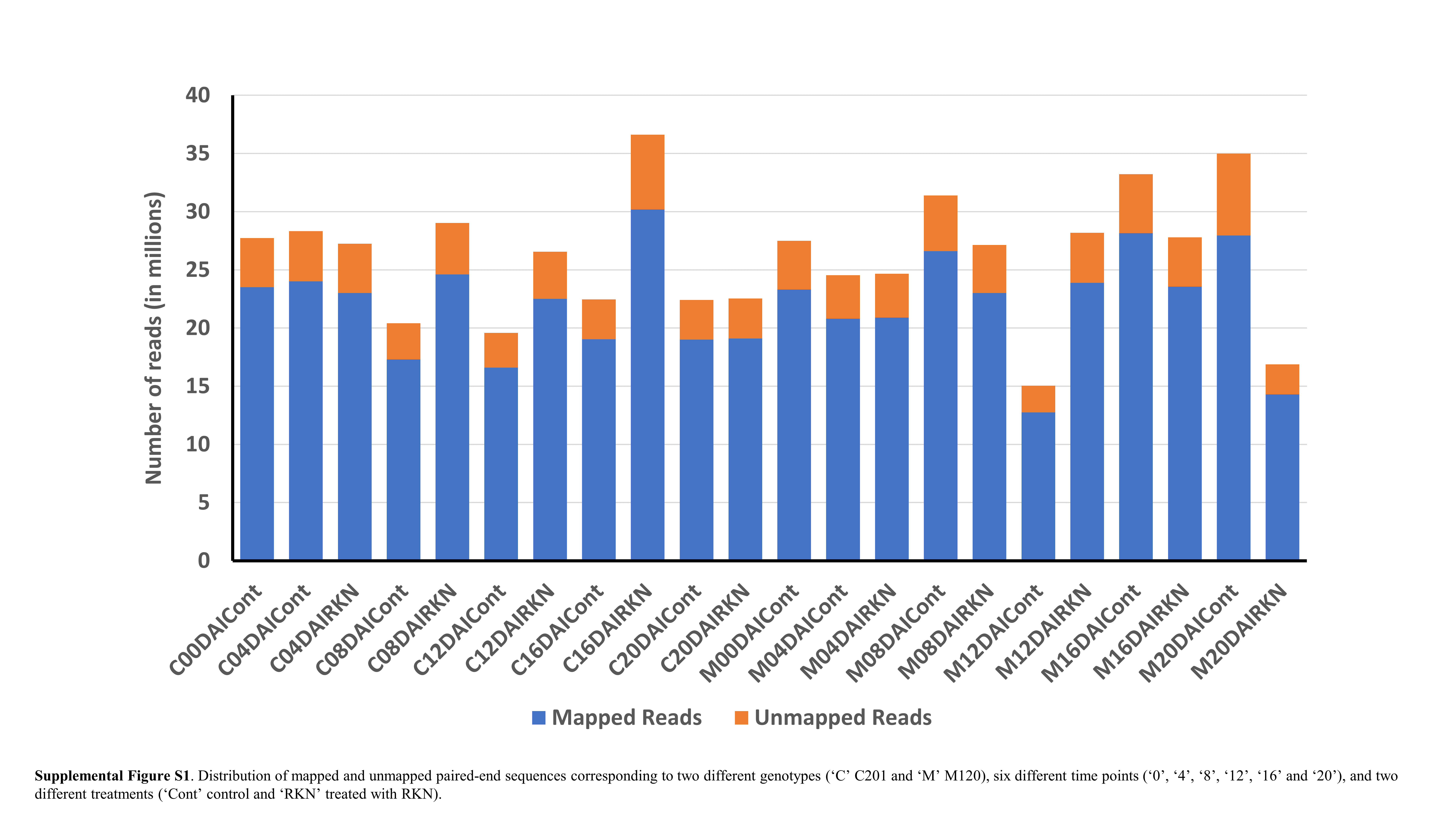

Supplement: Supplementary file 11 — Supplementary Material 11: Supplemental Figure S1. Distribution of mapped and unmapped paired-end sequences corresponding to two different genotypes (‘C’ C201 and ‘M’ M120), six different time points (‘0’, ‘4’, ‘8’, ‘12’, ‘16’ and ‘20’), and two different treatments (‘Cont’ control and ‘RKN’ treated with RKN). [file 12864_2025_11339_MOESM11_ESM.tiff]

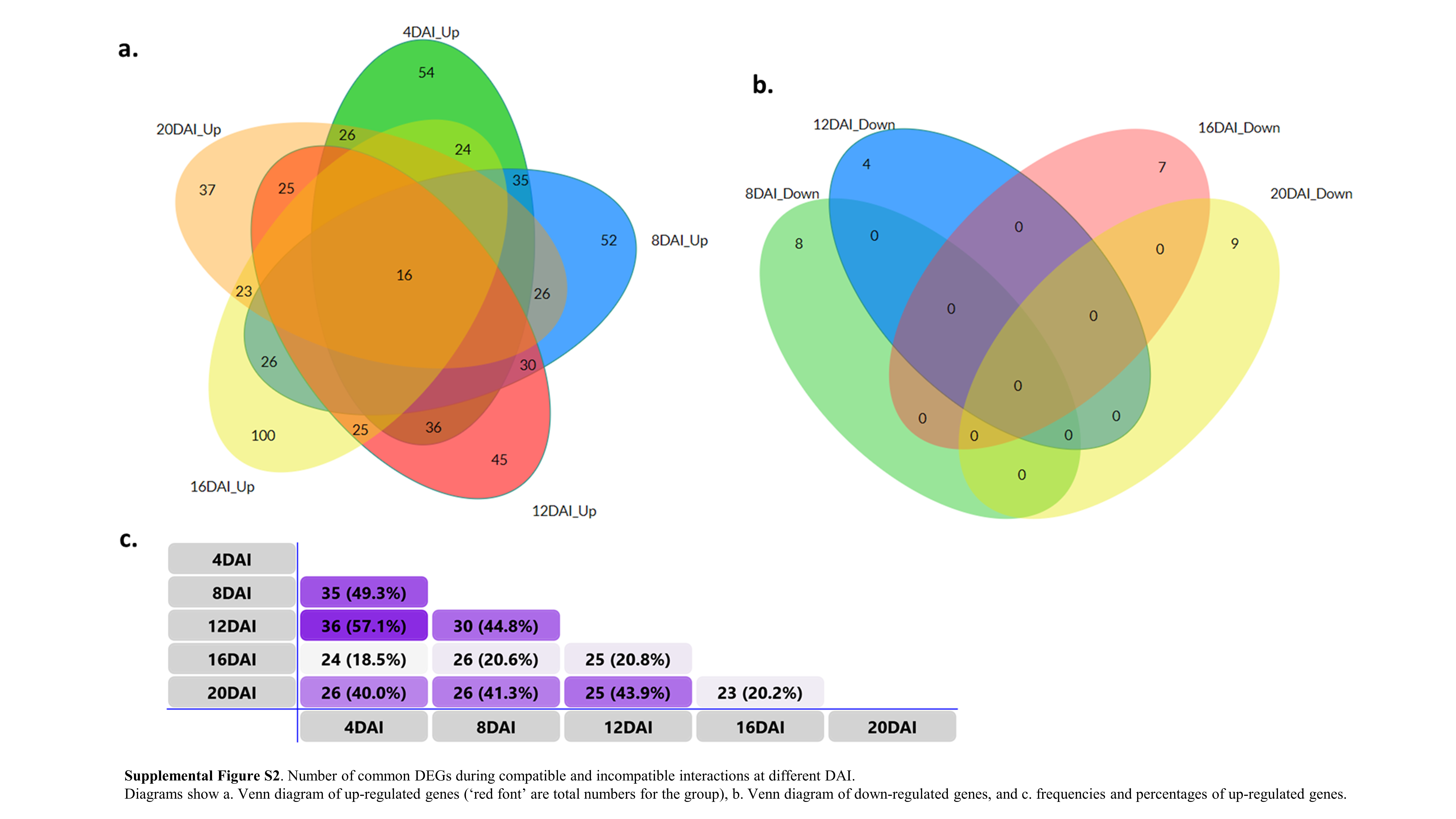

Supplement: Supplementary file 12 — Supplementary Material 12: Supplemental Figure S2. Number of common DEGs during compatible and incompatible interactions at different DAI. Diagrams show a. Venn diagram of upregulated genes (‘red font’ are total numbers for the group), b. Venn diagram of downregulated genes, and c. frequencies and percentages of upregulated genes. [file 12864_2025_11339_MOESM12_ESM.tiff]

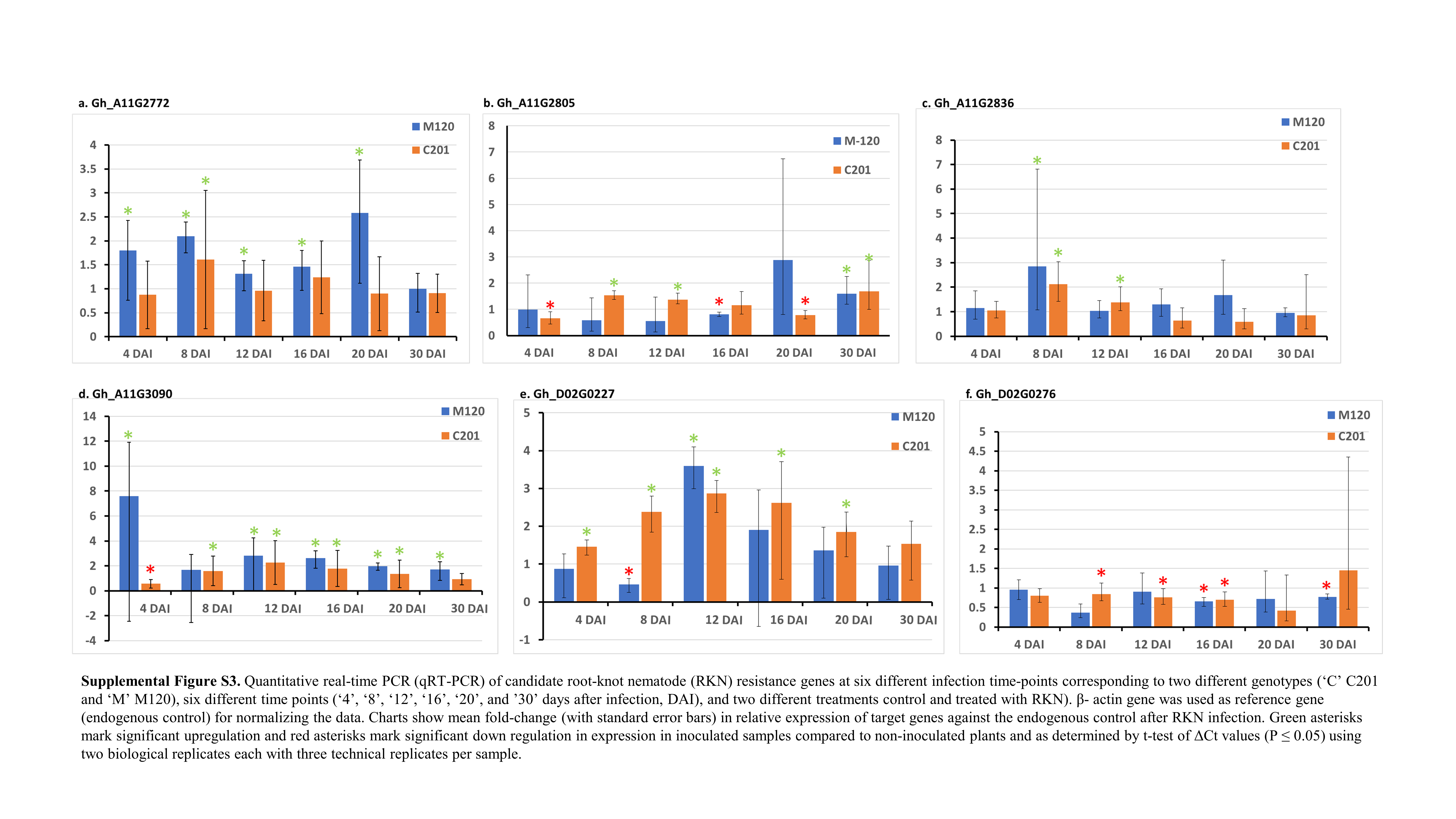

Supplement: Supplementary file 13 — Supplementary Material 13: Supplemental Figure S3. Quantitative real-time PCR (qRT-PCR) of candidate root-knot nematode (RKN) resistance genes at six different infection time points corresponding to two different genotypes (‘C’ C201 and ‘M’ M120), six different time points (‘4’, ‘8’, ‘12’, ‘16’, ‘20’, and ’30’ days after infection, DAI), and two different treatments control and treated with RKN). β- actin gene was used as reference gene (endogenous control) for normalizing the data. Charts show mean fold-change (with standard error bars) in relative expression of target genes against the endogenous control after RKN infection. Green asterisks mark significant upregulation, and red asterisks mark significant down regulation in expression in inoculated samples compared to non-inoculated plants and as determined by t-test of ΔCt values (P ≤ 0.05) using two biological replicates each with three technical replicates per sample. [file 12864_2025_11339_MOESM13_ESM.tiff]

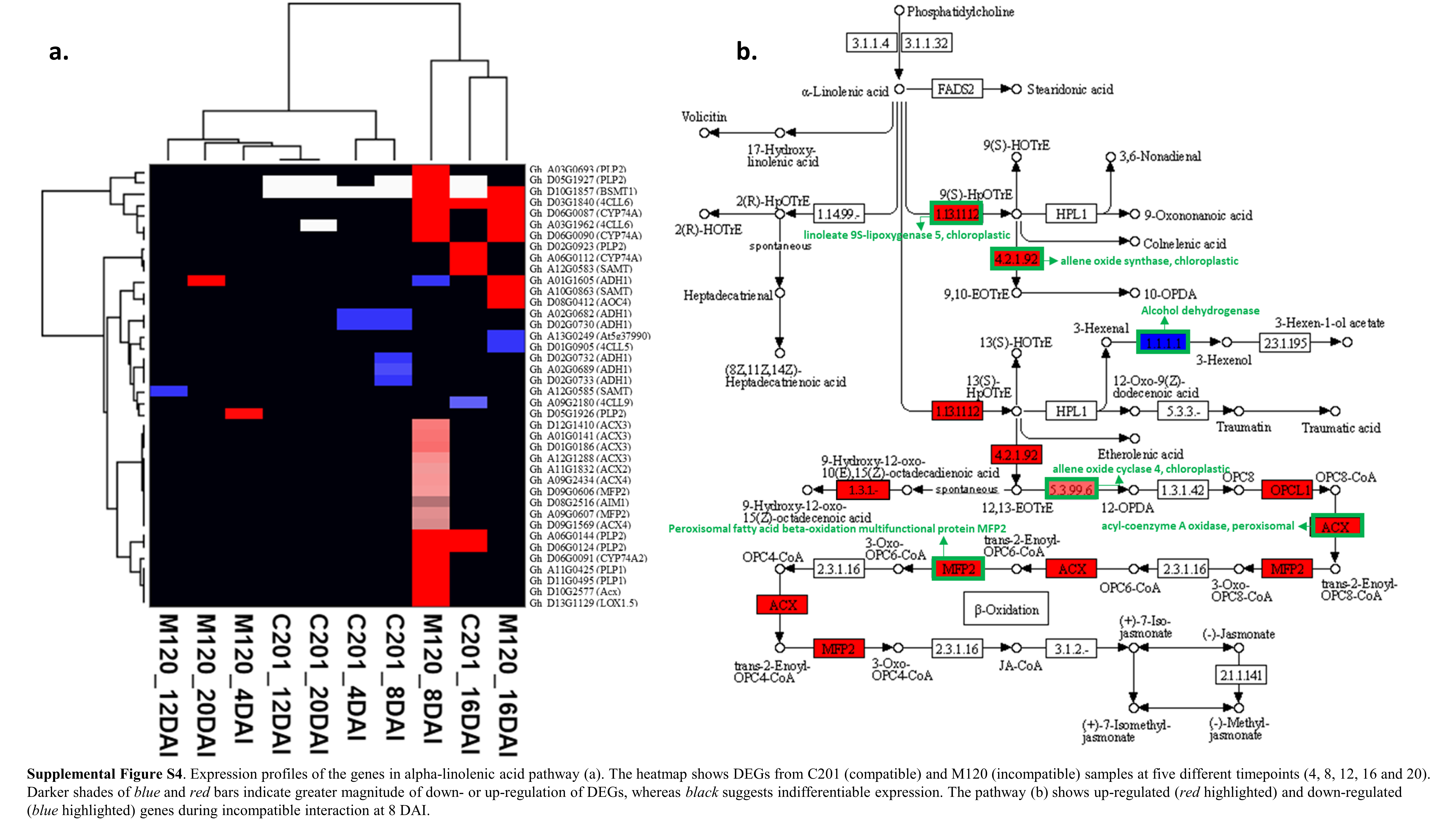

Supplement: Supplementary file 14 — Supplementary Material 14: Supplemental Figure S4. Expression profiles of the genes in alpha-linolenic acid pathway during compatible and incompatible interactions. The heat map (a) shows DEGs from C201 (compatible) and M120 (incompatible) samples at five different time points (4, 8, 12, 16 and 20). Darker shades of blue and red bars indicate greater magnitude of down- or upregulation of DEGs, whereas black suggests indifferentiable expression. The pathway (b) shows upregulated (red highlighted) and downregulated (blue highlighted) genes during incompatible interaction at 8 DAI. [file 12864_2025_11339_MOESM14_ESM.tiff]

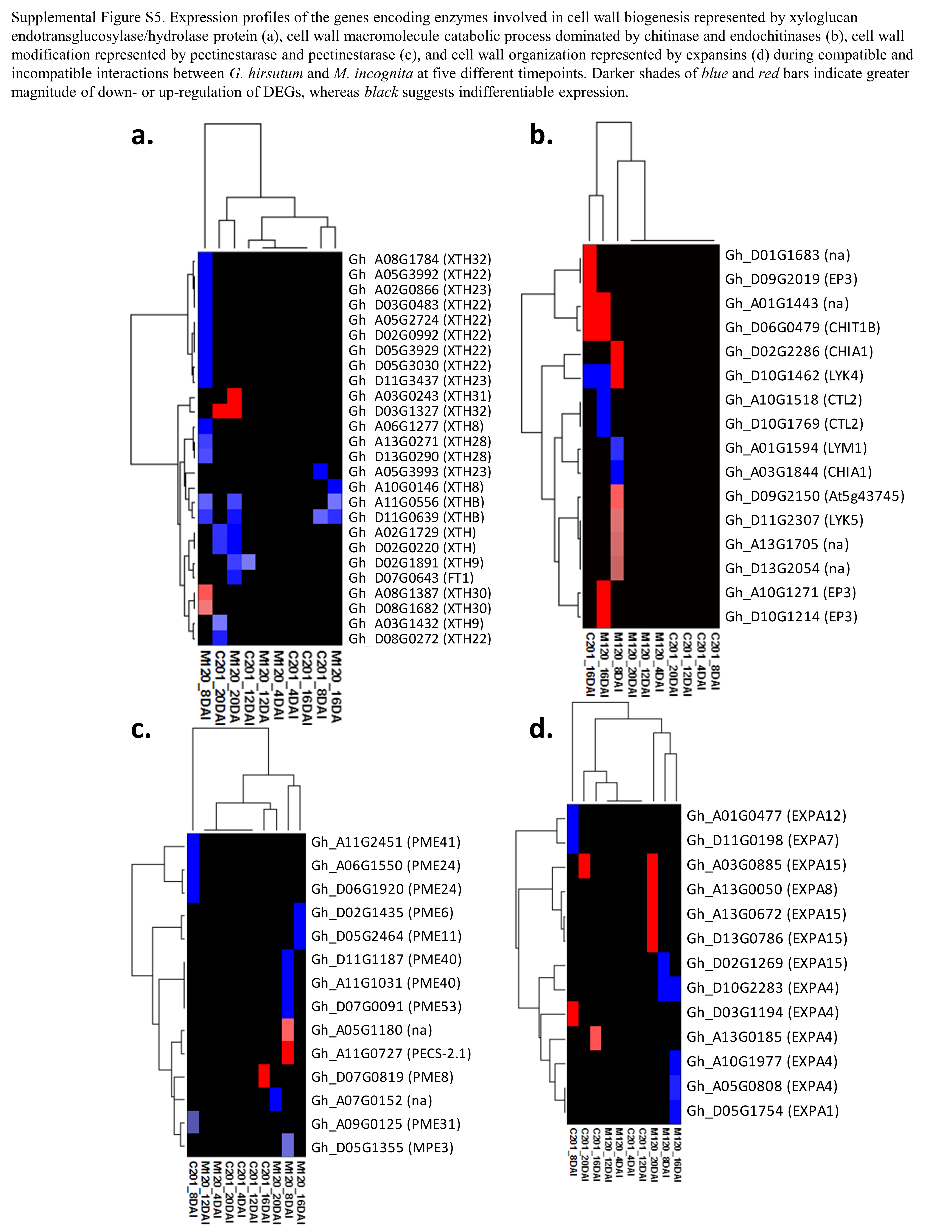

Supplement: Supplementary file 15 — Supplementary Material 15: Supplemental Figure S5. Expression profiles of the genes encoding enzymes involved in cell wall biogenesis represented by xyloglucan endotransglucosylase/hydrolase protein (a), cell wall macromolecule catabolic process dominated by chitinase and endochitinases (b), cell wall modification represented by pectinestarase (c), and cell wall organization represented by expansins (d) during compatible and incompatible interactions between G. hirsutum and M. incognita at five different time points. Darker shades of blue and red bars indicate greater magnitude of down- or upregulation of DEGs, whereas black suggests indifferentiable expression. [file 12864_2025_11339_MOESM15_ESM.tiff]

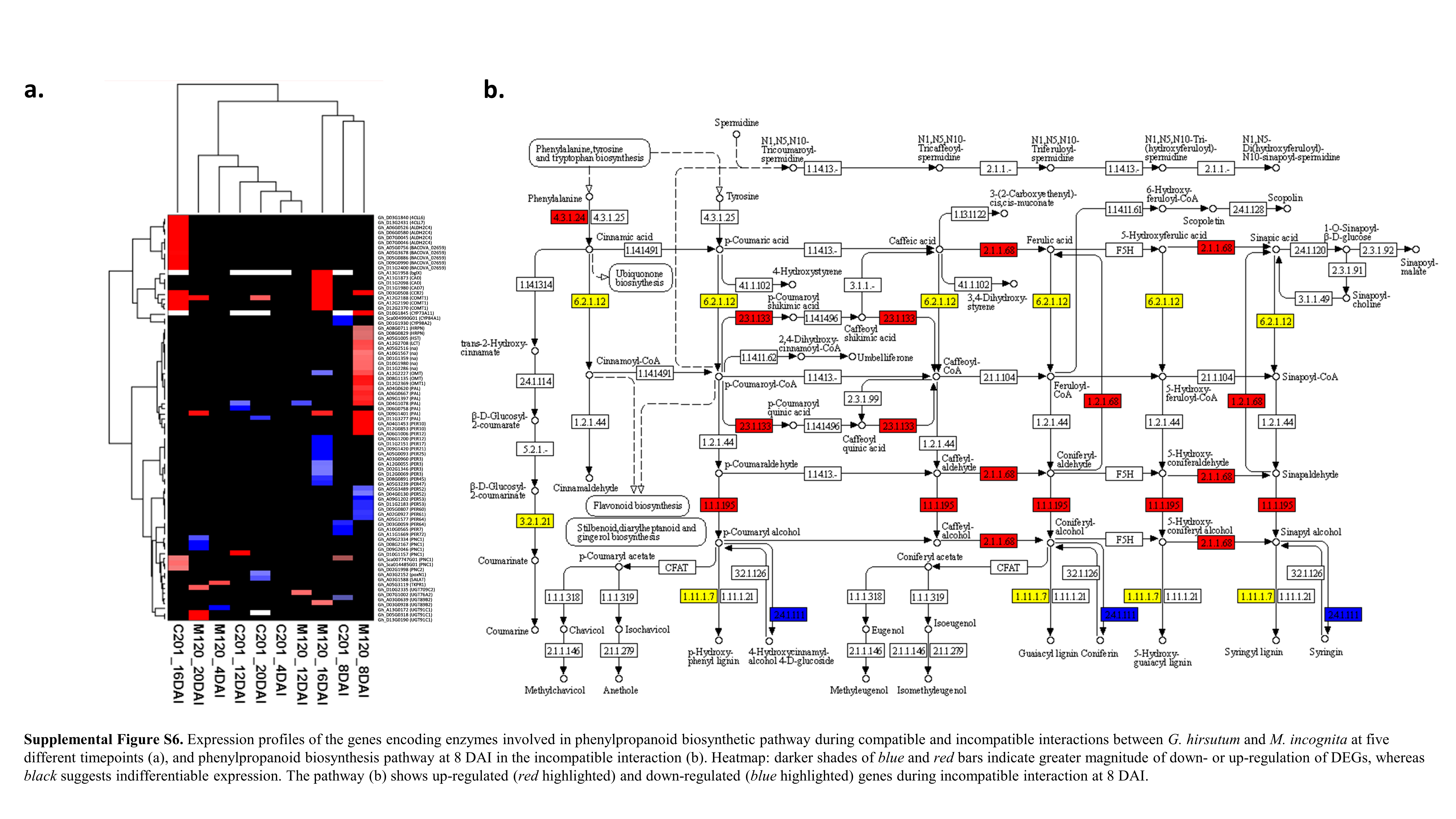

Supplement: Supplementary file 16 — Supplementary Material 16. Supplemental Figure S6. Expression profiles of the genes encoding enzymes involved in phenylpropanoid biosynthetic pathway during compatible and incompatible interactions between G. hirsutum and M. incognita at five different time points (a), and phenylpropanoid biosynthesis pathway at 8 DAI in the incompatible interaction (b). Heatmap: darker shades of blue and red bars indicate greater magnitude of down- or upregulation of DEGs, whereas black suggests indifferentiable expression. The pathway (b) shows upregulated (red highlighted) and downregulated (blue highlighted) genes during incompatible interaction at 8 DAI. [file 12864_2025_11339_MOESM16_ESM.tiff]

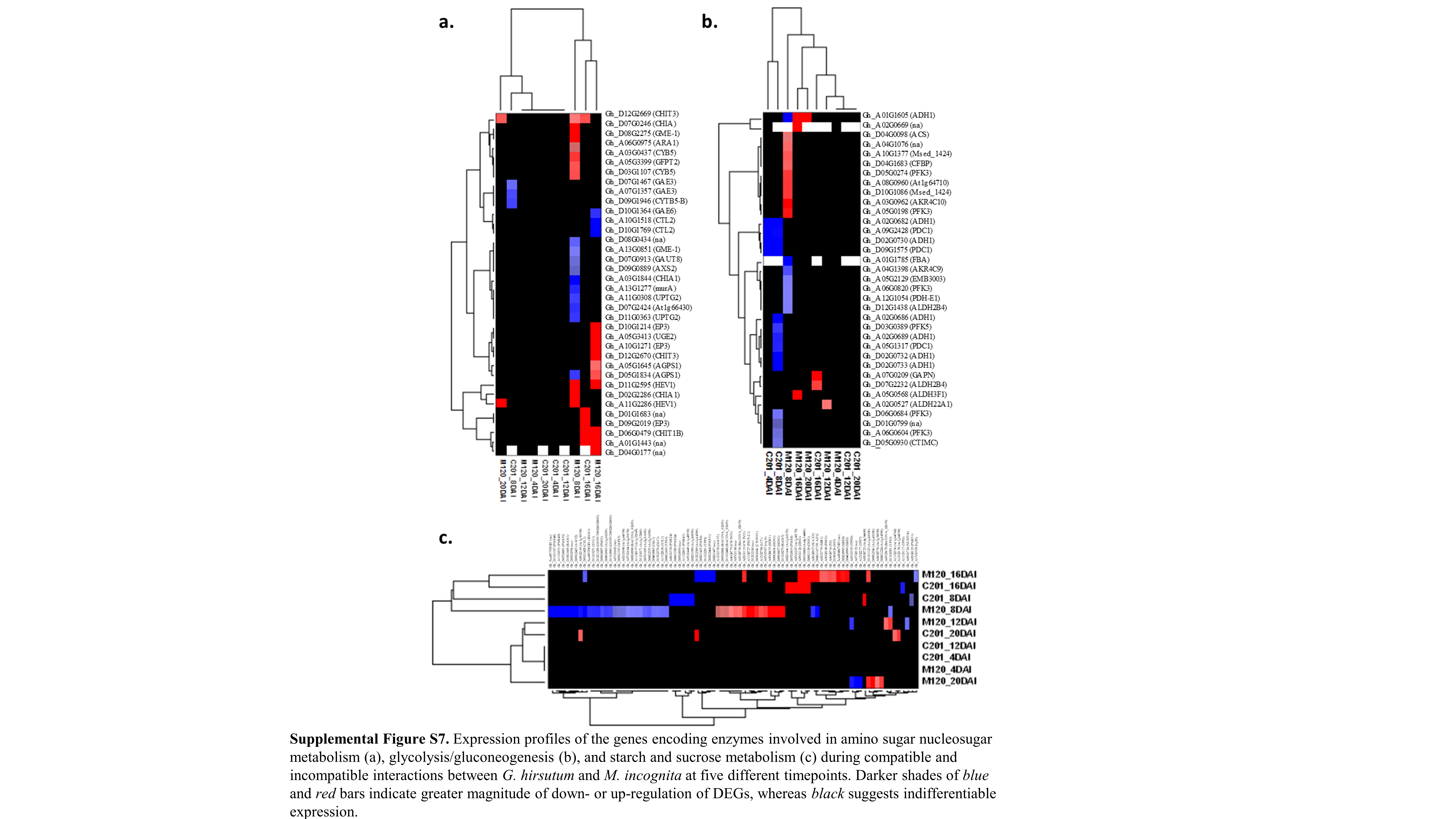

Supplement: Supplementary file 17 — Supplementary Material 17: Supplemental Figure S7. Expression profiles of the genes encoding enzymes involved in amino sugar nucleosugar metabolism (a), glycolysis/gluconeogenesis (b), and starch and sucrose metabolism (c) during compatible and incompatible interactions between G. hirsutum and M. incognita at five different time points. Darker shades of blue and red bars indicate greater magnitude of down- or upregulation of DEGs, whereas blacksuggests indifferentiable expression. [file 12864_2025_11339_MOESM17_ESM.tiff]

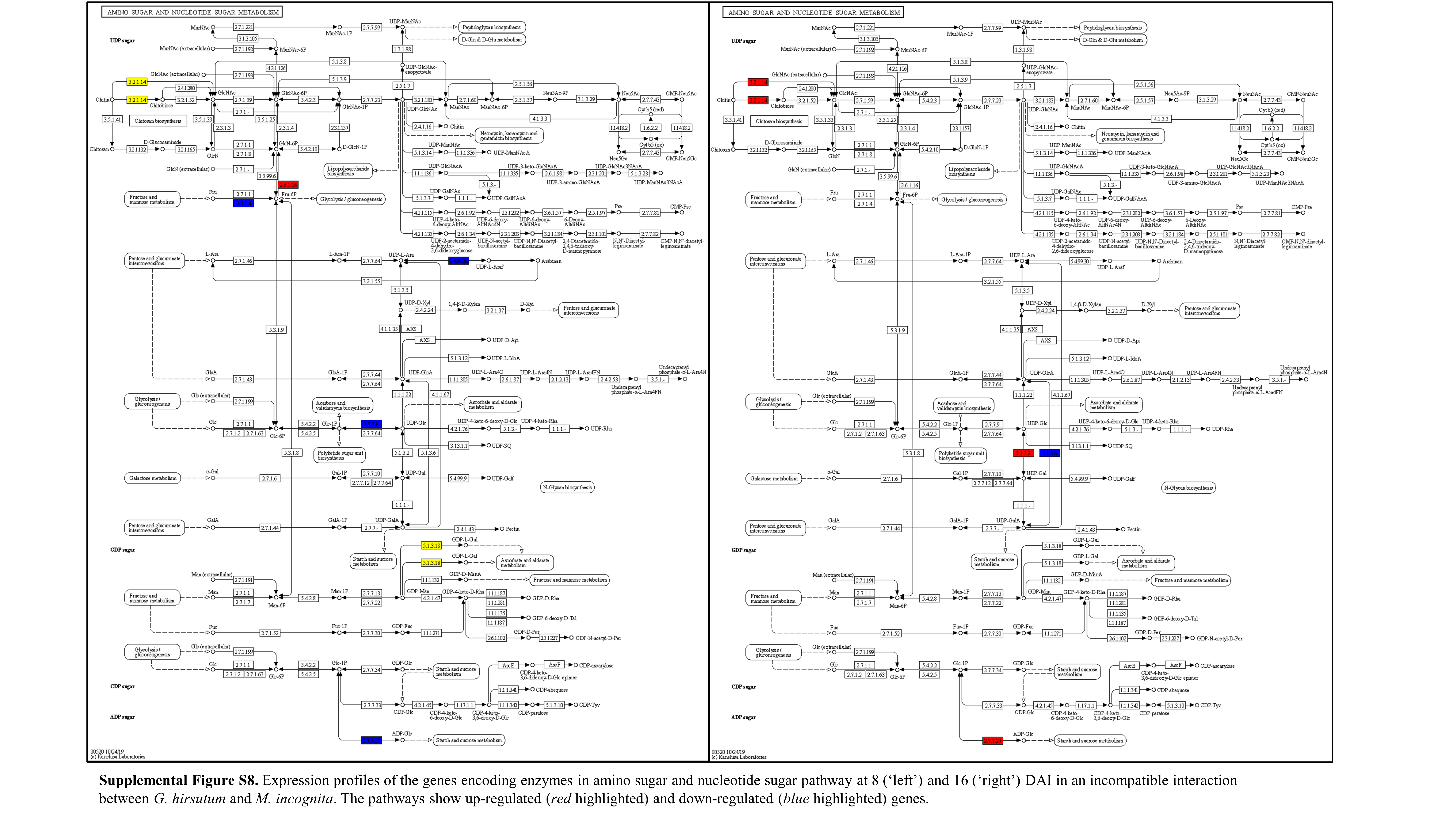

Supplement: Supplementary file 18 — Supplementary Material 18: Supplemental Figure S8. Expression profiles of the genes encoding enzymes in amino sugar and nucleotide sugar pathway at 8 (‘left’) and 16 (‘right’) DAI in an incompatible interaction between G. hirsutum and M. incognita. The pathways show upregulated (red highlighted) and downregulated (blue highlighted) genes. [file 12864_2025_11339_MOESM18_ESM.tiff]

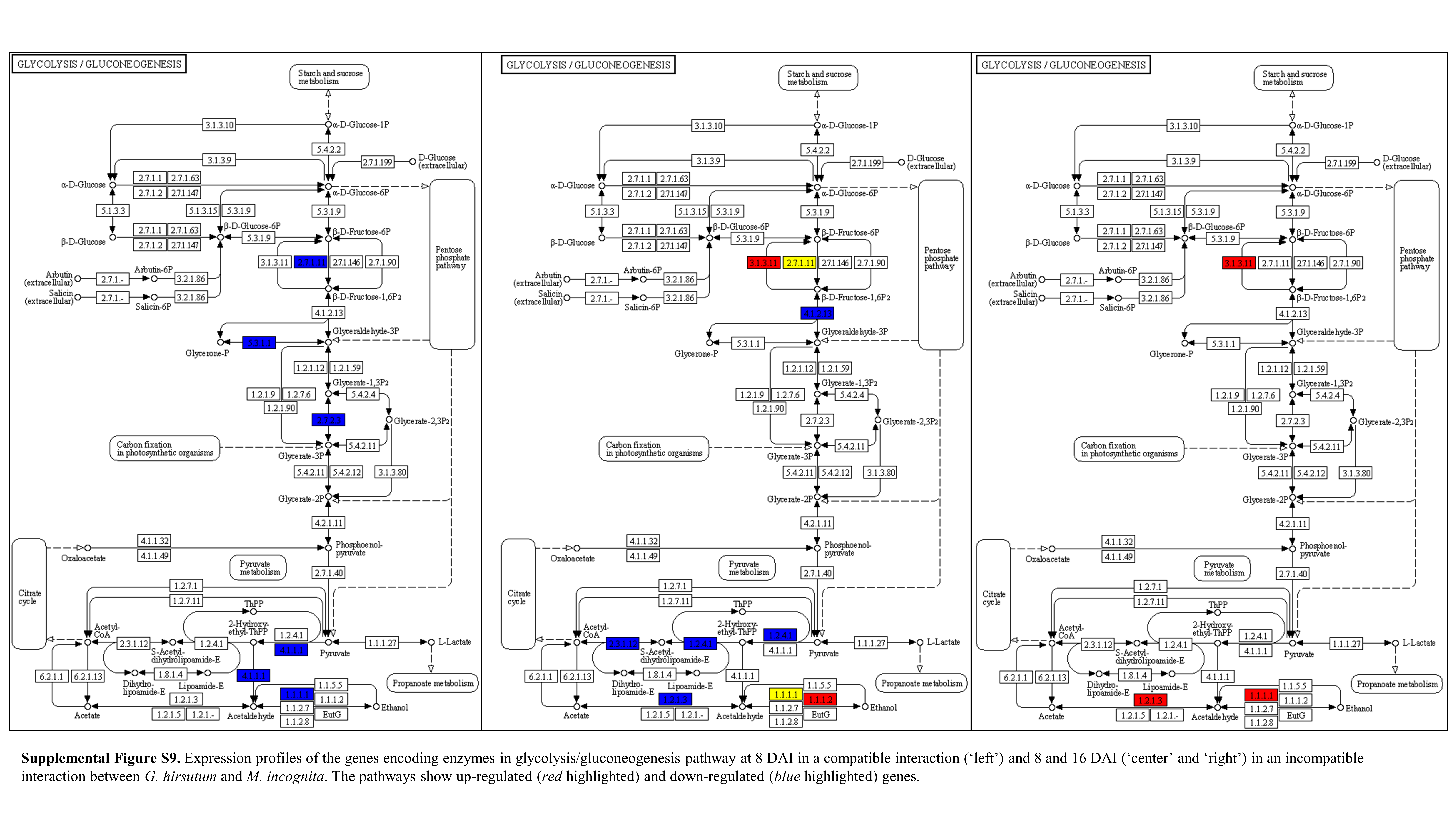

Supplement: Supplementary file 19 — Supplementary Material 19: Supplemental Figure S9. Expression profiles of the genes encoding enzymes in glycolysis/gluconeogenesis pathway at 8 DAI in a compatible interaction (‘left’) and 8 and 16 DAI (‘center’ and ‘right’) in an incompatible interaction between G. hirsutum and M. incognita. The pathways show upregulated (red highlighted) and downregulated (blue highlighted) genes. [file 12864_2025_11339_MOESM19_ESM.tiff]

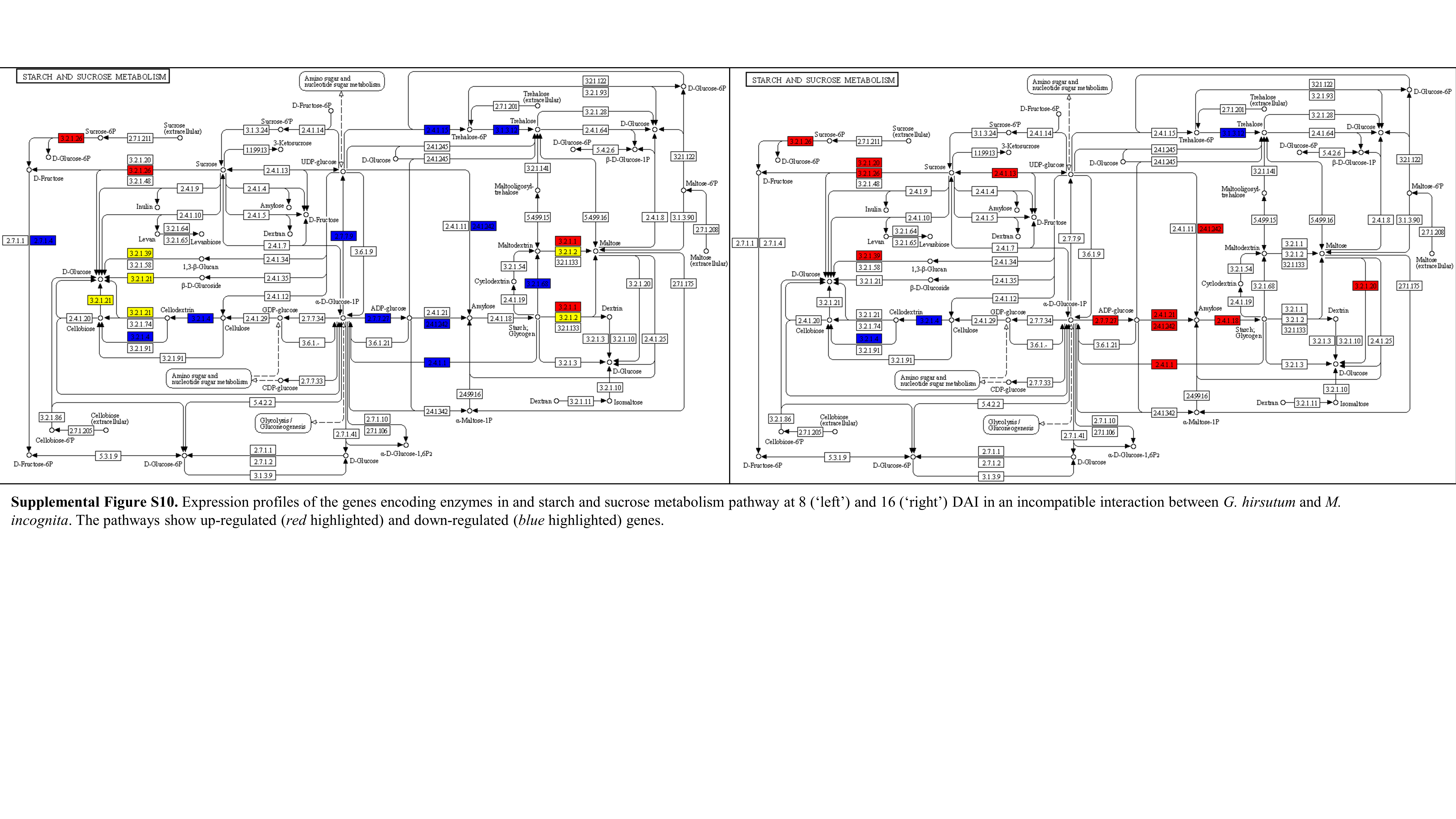

Supplement: Supplementary file 20 — Supplementary Material 20: Supplemental Figure S10. Expression profiles of the genes encoding enzymes in starch and sucrose metabolism pathway at 8 (‘left’) and 16 (‘right’) DAI in an incompatible interaction between G. hirsutum and M. incognita. The pathways show upregulated (red highlighted) and downregulated (blue highlighted) genes. [file 12864_2025_11339_MOESM20_ESM.tiff]
